# Impact of double split slot geometry on aerodynamic performance of modified airfoil for wind turbine blades

**DOI:** 10.1038/s41598-025-12174-5

**Published:** 2025-08-11

**Authors:** Dipankar Sarkar, Anal Ranjan Sengupta, Prabhu Paramasivam, Bhaskor Jyoti Bora, Natrayan Lakshmaiya, Paragmoni Kalita, Abinet Gosaye Ayanie, Praveen Kumar Kanti

**Affiliations:** 1 Rajiv Gandhi Institute of Petroleum Technology Bengaluru Campus, Bengaluru, Karnataka 562165 India; 2 Department of Mechanical Engineering, JIS College of Engineering, Kalyani, West Bengal 7421235 India; 3 Centre for Research Impact & Outcome, Chitkara University Institute of Engineering and Technology, Chitkara University, Rajpura, Punjab 140401 India; 4 Rajiv Gandhi Institute of Petroleum Technology Sivasagar Campus, Sivasagar, Assam 785697 India; 5 Department of Mechanical Engineering, Saveetha School of Engineering, SIMATS, Chennai, Tamil Nadu 602105 India; 6 Department of Mechanical Engineering, School of Engineering, Tezpur University, Assam, 784028 India; 7 Department of Mechanical Engineering, Adama Science and Technology University, 2552 Adama, Ethiopia; 8 University Center for Research & Development (UCRD), Chandigarh University, Mohali, Punjab India

**Keywords:** Slotted airfoil, Passive flow control, Flow separation, Wind turbine blade, CFD, Aerospace engineering, Mechanical engineering

## Abstract

Introducing a slot into an airfoil is a passive flow control technique that enhances aerodynamic performance by manipulating the boundary layers of fluid flow. This study investigates the aerodynamic performance of a novel double-split slot design using the NACA 0018 airfoil through a detailed 2D steady-state numerical analysis. A parametric study was conducted to evaluate the influence of key design parameters, including slot outlet location, outlet width, and wedge element length, on the force coefficients and the flow structure around the airfoil. Results demonstrate that the double-split slot effectively weakened the flow separation on the suction side, at moderate to higher angles of attack (15° ≤ α ≤ 30°). The optimal slot configuration achieved a lift coefficient (*C*_*L*_) improvement of 118% and a drag coefficient (*C*_*D*_) reduction of 49% compared to the baseline clean airfoil. Slot configurations with outlets positioned closer to the leading edge (LE), wider outlet widths, and longer split channels displayed improved performance by preventing flow detachment. Most double-split slots delayed flow separation by up to 10° in AOA. Overall, slotted airfoils demonstrated superior performance over clean airfoils at higher AOAs, making them particularly beneficial for vertical-axis wind turbine blade applications.

## Introduction

The growing global energy demand and the necessity to reduce carbon emissions have accelerated the shift toward renewable energy sources. Among these sources, wind energy has become the second-largest contributor, accounting for its significant share of the global renewable energy capacity due to its availability and viability. The engineering applications of wind energy conversion are predominantly focused on wind turbines. Despite the increasing adoption of wind energy, several technical challenges persist, particularly in enhancing wind turbine productivity and annual energy production (AEP). One major challenge is the flow separation around the turbine blades, which reduces efficiency and leads to stall formation, unsteady loads, structural damage, and noise generation.

Reducing flow separation and unsteady flow patterns over turbine blades is essential and can be achieved by boosting energy mixing within boundary layers. Generally, two primary flow control strategies—active flow control (AFC) and passive flow control (PFC)—are commonly used to maintain flow attachment to blade surfaces. AFC involves using moving components that actively counteract flow separation by consuming external energy. Advanced AFC technologies include plasma actuators^1–4^, flaps^5–7^, variable pitching^8–10^, suction/blowing slots^11^, synthetic jets^12^ and blade morphing^13–16^. However, AFCs are often complex, requiring precise control loops or microcontrollers for effective operation.

In contrast, PFC uses static components or geometric modifications to the existing structure to manage the flow. These devices are typically fixed, less customizable, inexpensive, and easy to install, but require precise orientation and positioning for optimal performance. Incorrect placement can lead to reduced effectiveness or adverse outcomes^17^.

Recent studies have demonstrated that PFC techniques significantly enhance the aerodynamic performance of both isolated airfoils and vertical-axis wind turbine (VAWT) configurations^18^. Effective PFC methods include the vortex generator (VG)^19–24^, guide vanes^25^, leading-edge protuberances^26–32^, leading-edge serrations^15,16,33,34^, leading edge slats^35^, leading-edge micro cylinder^36,37^, Gurney flaps^15,16,38^, slots^13,17,39–49^, cavities^50^, Yousefi^51^. To maximize aerodynamic performance, a wind turbine airfoil must satisfy the following criteria, as outlined by^52^:

- Achieve a high lift coefficient (*C*_*L*_)
- Maintain a high glide ratio or lift-over-drag coefficient (*C*_*L*_*/C*_*D*_)
- Perform effectively under complex wind conditions across a wide operating range.

Identifying an optimal airfoil geometry that delivers consistent peak performance across a broad operational range remains a significant challenge. Many PFC methods rely on complex principles, limiting their practicality and yielding minimal performance gains in real-world applications. However, slotted airfoils have emerged as a promising solution, effectively suppressing flow separation and enhancing aerodynamic performance^41,49,53,54^.

The slot concept for airfoils was first introduced by Handley^55^ for aircraft wings and later refined by Lachmann^56^ for high-lift configurations with multiple slotted wings. Weick and Shortal^57^, analysed optimal slot and flap combinations on a Clark-Y wing, while Weinzinger and Shortal^58^ tested 100 slot layouts, varying slot gap, width, and depth to improve performance. Drela^59,60^ advanced the concept using multi-point optimisation for high-lift airfoils. Ramzi and AbdErrahmane^61^ demonstrated that incorporating a slot in a cascade to postpone boundary layer separation reduced the loss coefficient by 28%. Narsipur et al.^46^ investigated the impact of multielement airfoil orientations on efficiency but noted that the optimal configuration remains undefined due to geometric complexity.

The aerodynamic performance of slotted wind turbine blades was initially tested under isolated static conditions. Xie et al.^49^ experimentally and computationally demonstrated that slotted blades increase lift and reduce drag at high angles of attack (AOA) by disrupting large separation zones. Ibrahim et al.^62^ and Beyhaghi et al.^53^ introduced leading edge (LE) slots to direct flow to the pressure surface, increasing static pressure. Beyhaghi et al.^42^ optimized slotted airfoil geometries using the response surface method (RSM), achieving significant aerodynamic improvements. Combining slotted airfoils with other passive flow control techniques, such as serrated trailing edges, further enhanced lift across various AOAs^44^. However, at large stall conditions, the aerodynamic performance becomes less sensitive to slot geometrical parameters^63,64^.

Recent studies have concentrated on developing slot layouts to enhance airfoil performance. Ni et al.^47^ introduced a novel curvilinear slot design through numerical and experimental tests, achieving simultaneous improvements in *C*_*L*_ and *C*_*D*_, with strong agreement between CFD results and experimental data. This slot design was later applied to hydrofoils by Ni et al.^54^. Belamadi et al.^41^ have numerically analysed a slotted NREL S809 airfoil, providing a comprehensive analysis of slot geometry. Their findings revealed that convergent slots mitigated flow separation more effectively than uniform slots, achieving a higher lift-over-drag ratio (*C*_*L*_*/C*_*D*_) across a broader AOA range. Moshfeghi et al.^17^ developed a unique double-split slot design with a single inlet and two outlet exits by combining two single slots, resulting in improved *C*_*L*_ at AOAs between 17 and 22°. Bhavsar et al.^43^ modelled five convergent-type slots on the thick DU-99-W-405 airfoil, further enhancing aerodynamic efficiency. Additionally, slots have been shown to reduce noise generated by turbine blades^40^. Cui et al.^65^ investigated various slot geometries in hydrofoils and reported that slots mitigate cavitation potential. They found that slot outlets near the trailing edge (TE) increase load fatigue, while those positioned closer to the LE reduce force oscillations and improve hydrodynamic performance.

The use of slotted airfoils in VAWT systems has been explored for the potential advantages they offer. Mohamed et al.^45^ simulated a slotted NACA 0018 airfoil under isolated conditions and later applied the optimized slot case to a VAWT system. The results demonstrated improved self-starting capability at low tip-speed ratios (TSRs) and increased overall efficiency compared to a baseline VAWT. Abdolahifar and Karimian^39^ suggested that taller slotted blades in VAWT systems require sufficiently wider slot inlets and outflows to effectively suppress flow separation. Additionally,^63^ strategically placing the slot outlet downstream on the pressure side helps mitigate pre-stall effects, enhancing aerodynamic performance. Few previous investigations on Slotted airfoils are summarized in Table 1.

**Table 1:** Previous investigations on Slotted airfoils.

Authors	Airfoil profile	Study object	Slot description	Findings
Xie et al.^49^	S809	Static airfoil	Uniform slot	At higher AOA performed better than non-slotted airfoil The large separated area turns into small vortices
Belamadi et al.^41^	NREL S809	Static airfoil	Uniform and convergent slot	The slotted airfoil outperformed the baseline airfoil at higher AOA but at the expense of increased drag at lower AOA A convergent slot is preferred over a uniform slot Slots with higher slop performed better at high AOA
Moshfeghi et al.^17^	NREL S809	Static airfoil	Double-split slot	The double-split design has performed better at the higher AoA 17° < AOA < 22°, whereas acts like a single slot design at lower AoA
Bhavsar et al.^43^	DU-99-W-405	Static airfoil	Convergent slot	A thick airfoil is better at seizing unsteady flow behaviour The boundary layer is better suppressed when the slot outlet is closer to the separation point
Hongpeng et al.^44^	S814	Static airfoil	Slot and TE extension	The combination of two PFC techniques has actively countered the performance loss issues at higher and lower AoA jointly
Beyhaghi et al.^53^	NACA 4412	Static airfoil	Leading edge to Pressure side slot	By positioning the slot outlet at the pressure side, the blade’s aerodynamic performance is improved by creating a greater pressure difference between the airfoil’s two sides
Beyhaghi et al.^42^	NACA 4412	Static airfoil	Leading edge to Pressure side slot	The influence of the geometrical parameter of the slot on lift force and LoD was observed positively Robustness of the DoE and Optimization confirmed for this type of application
Ni et al.^47^	NACA 634,021	Static airfoil	Converging curved slot	The slot width should not be too wide to gain enough momentum to suppress the separation At mid-stall and post-stall regimes performance improved, but the low AOA (α < 11°) range still has a low lift
Ni et al.^54^	NACA 634,021	Static airfoil	Converging curved slot with Leading edge Tubercles	Perform better at moderate stall and post-stall region A periodic vortex formation was observed in the presence of tubercles
Akhter et al.,^13^	. NREL Phase-VI Blade_(S809)	Static airfoil	Inclined convergent slot	Torque and thrust increased at low wind speed (≤ 7 m/s) Separation, reverse flow, tip loss due to vortices and wake profile is reduced and shortened
Nia et al. (Bakhtiari^66^)	DU-97-W-300	Static airfoil	Slot and VGs	The combined PFC approach has shown higher lift values in all operating ranges compared to the slotted approach alone
Akhter et al.^40^	S809	Static airfoil	Inclined convergent slot	Enhancement in aerodynamic performance, whereas, reduction in far-field noise is observed from Aero-acoustic analyses
Abdolahifar and Karimian^39^	NACA 0021	3D H-Darrieus turbine (single blade)	Non-uniform converging curvilinear slot	An increase in the height of the blade necessitates a wider slot to mitigate a higher volume of flow separation A slotted blade increases the starting torque while weakening the dynamic stall at low TSR

Despite advancements in wind turbine technology, airfoil performance in the post-stall region remains suboptimal, particularly in controlling flow separation. Studies have shown that single contracting non-uniform slots in airfoils significantly improve performance, motivating further research into multi-element airfoil designs. To the best of the authors’ knowledge, no research has explored optimizing symmetrical airfoil performance using a slot with a single inlet and double outlets, featuring a converging flow path at AOAs. This study aims to develop a 2D numerical model of a double-split slotted airfoil under isolated static conditions to analyse the effects of slot geometry on aerodynamic performance. The focus is on aerodynamic behaviour influenced by slot geometry variations, without addressing the detailed flow physics in depth. The findings are expected to provide valuable new insights for the wind communities. The paper begins with an overview and literature review of slotted airfoils, followed by a detailed explanation of the numerical methodology. It then presents CFD simulation results and discussions, concluding with key findings and recommendations for future research.

## Numerical setup and methodology

### Slot geometry

**Fig. 1:**
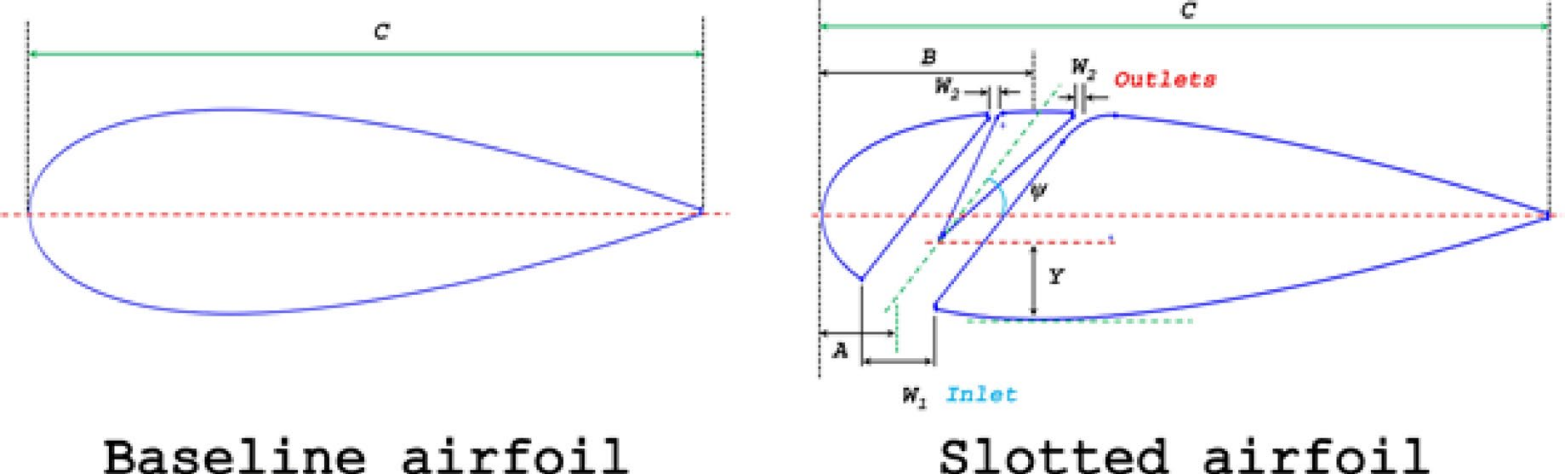
Schematic diagram of (**a**) Baseline airfoil and (**b**) Double-split slotted airfoil.

The study shows that symmetrical airfoils perform better over a wide range of AOAs, making them ideal for VAWTs, which experience dynamic changes in relative wind direction. In contrast, asymmetrical airfoils excel under static conditions, making them more suitable for HAWTs, which predominantly encounter unidirectional flow^67,68^. Previous research has chiefly incorporated slots into asymmetrical airfoils. The NACA 0018 airfoil was selected for its advantageous characteristics in VAWT applications. A 2D numerical model was developed using the NACA 0018 profile as the baseline^69^ as the baseline case (Fig. 1). Experimental data on VAWTs with NACA 0018 airfoil is widely abundant^8,11,70^.

**Fig. 2:**
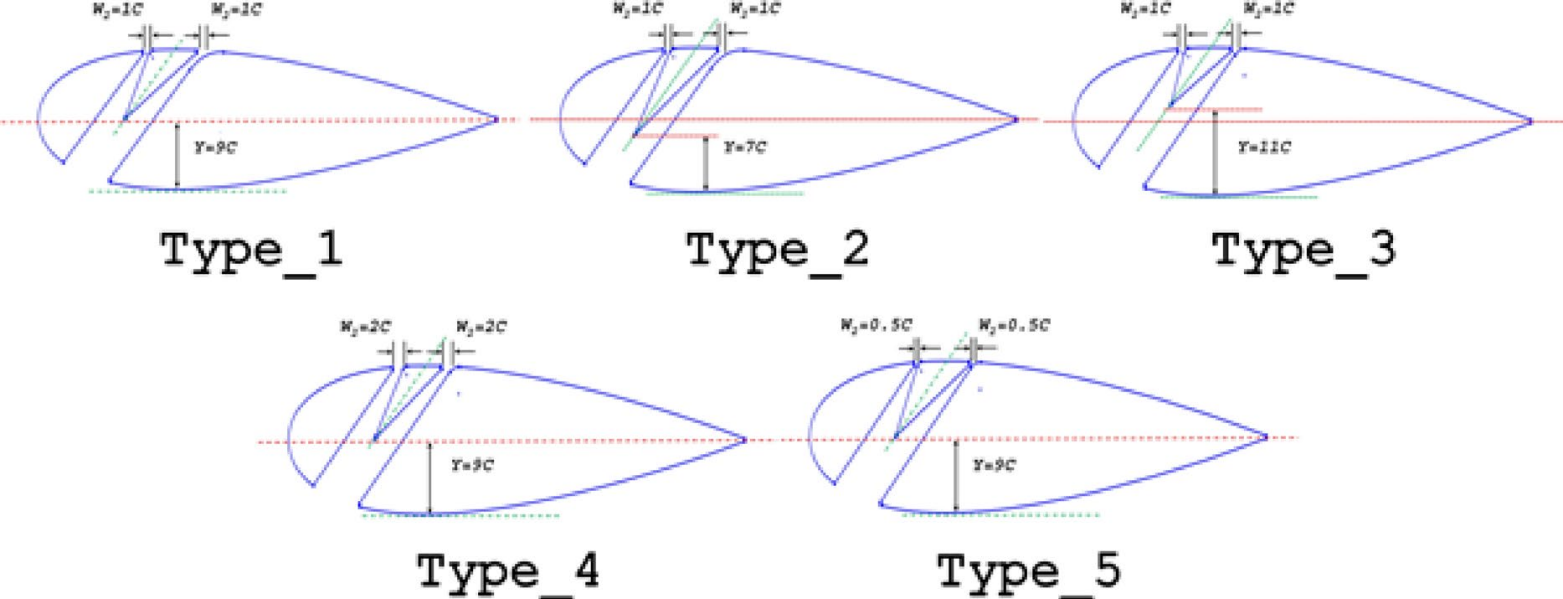
Different layouts of a double-split slotted airfoil and geometrical entities.

The airfoil has a chord length of *C* = 0.47 m and a squared TE, as described by Mohamed et al.^45^. Five slot types were defined based on the outlet width (*W*_*2*_) and the distance from the wedge tip (2nd airfoil element) to the location of the airfoil’s maximum bottom thickness (*Y*) Fig. 2. In all slot configurations, the slot’s centreline at the pressure surface (*A*) remains unchanged, while the inlet width (*W*_*1*_) increases as the centreline at the suction surface shifts toward the TE. Table 2 summarises the dimensions of these slot configurations.

**Table 2:** Details of slot configurations.

Category	Cases	Y (%C)	W_2_ (%C)		B (%C)	W_1_ (%C)	Ψ (°)
Type 1	T1_20C	9C		1	20	8.3	57.39
	T1_30C				30	9.97	38.71
	T1_40C				40	12.045	27.67
	T1_50C				50	14.3	20.51
	T1_60C				60	16.79	15.5
	T1_70C				70	19.65	11.79
Type 2	T2_20C	7C		1	20	8.3	57.39
	T2_30C				30	9.97	38.71
	T2_40C				40	12.045	27.67
	T2_50C				50	14.3	20.51
	T2_60C				60	16.79	15.5
	T2_70C				70	19.65	11.79
Type 3	T3_20C	11C		1	20	8.3	57.39
	T3_30C				30	9.97	38.71
	T3_40C				40	12.045	27.67
	T3_50C				50	14.3	20.51
	T3_60C				60	16.79	15.5
	T3_70C				70	19.65	11.79
Type 4	T4_T1_30C	9C		2	30	9.97	57.39
	T4_T1_40C	9C			40	12.045	38.71
	T4_T2_30C	7C			30	9.97	27.67
	T4_T2_40C	7C			40	12.045	20.51
	T4_T3_40C	11C			40	12.045	15.5
Type 5	T5_T1_30C	9C		0.5	30	9.97	57.39
	T5_T1_40C	9C			40	12.045	38.71
	T5_T2_30C	7C			30	9.97	27.67
	T5_T2_40C	7C			40	12.045	20.51
	T5_T3_40C	11C			40	12.045	15.5

### Turbulence modelling

The selection of an appropriate turbulence model is crucial for accurately analysing the flow behaviour. Following the works of Belamadi et al.^41^, Mohamed et al.^45^ and Abdolahifar and Karimian^39^, this study employed the realizable *k-ε* turbulence model within the RANS framework. This model provides better predictions of complex flow phenomena, including curvature, rotation, and separation on the airfoil surface, compared to the standard *k-ε* model^71,72^. The simulations were performed using the Ansys Fluent version 2022 R2 (22.2) software package. For the particular 2D steady-state incompressible problem, the model uses mass and momentum conservation (Eqs. 1, 2 and 3) with the modelled transport equations (Eqs. 4 and 5).

The continuity equation is,

1 $$\frac{\partial u}{\partial x}+\frac{\partial v}{\partial y}=0$$

and, x-momentum equation,

2 $$\rho \left(u\frac{\partial u}{\partial x}+v\frac{\partial u}{\partial y}\right)=-\frac{\partial P}{\partial x}+\mu \left(\frac{{\partial }^{2}u}{\partial {x}^{2}}+\frac{{\partial }^{2}u}{\partial {y}^{2}}\right)+\rho {g}_{x}$$

y-momentum equation,

3 $$\rho \left(u\frac{\partial v}{\partial x}+v\frac{\partial v}{\partial y}\right)=-\frac{\partial P}{\partial x}+\mu \left(\frac{{\partial }^{2}v}{\partial {x}^{2}}+\frac{{\partial }^{2}v}{\partial {y}^{2}}\right)+\rho {g}_{y}$$

Kinetic energy (*k*) and specific dissipation rate (*ε*) for the *k-ε* turbulence model are

4 $$\frac{\partial }{\partial t}\left(\rho k\right)+\frac{\partial }{\partial {x}_{j}}(\rho k{u}_{j})=\frac{\partial }{\partial {x}_{j}}\left[\left(\mu +\frac{{\mu }_{t}}{{\sigma }_{k}}\right)\frac{\partial k}{\partial {x}_{j}}\right]+{G}_{k}+{G}_{b}-\rho \varepsilon -{Y}_{M}+{S}_{k}$$

and

5 $$\frac{\partial }{\partial t}\left(\rho \varepsilon \right)+\frac{\partial }{\partial {x}_{j}}\left(\rho \varepsilon {u}_{j}\right)=\frac{\partial }{\partial {x}_{j}}\left[\left(\mu +\frac{{\mu }_{t}}{{\sigma }_{\varepsilon }}\right)\frac{\partial \varepsilon }{\partial {x}_{j}}\right]+{\rho C}_{1}{S}_{\varepsilon }-{\rho C}_{2}\frac{{\varepsilon }^{2}}{k+\sqrt{\upsilon \varepsilon }}+{C}_{1\varepsilon }\frac{\varepsilon }{k}{C}_{3\varepsilon }{G}_{b}+{S}_{\varepsilon }$$

where,

6 $${C_1} = {\text{max}}\left[ {0.43,\frac{\eta }{{\eta + 5}}} \right], = S\frac{k}{\varepsilon } , S = \sqrt {2{S_{ij}}{S_{ij}}}$$

### Computational setup

**Fig. 3:**
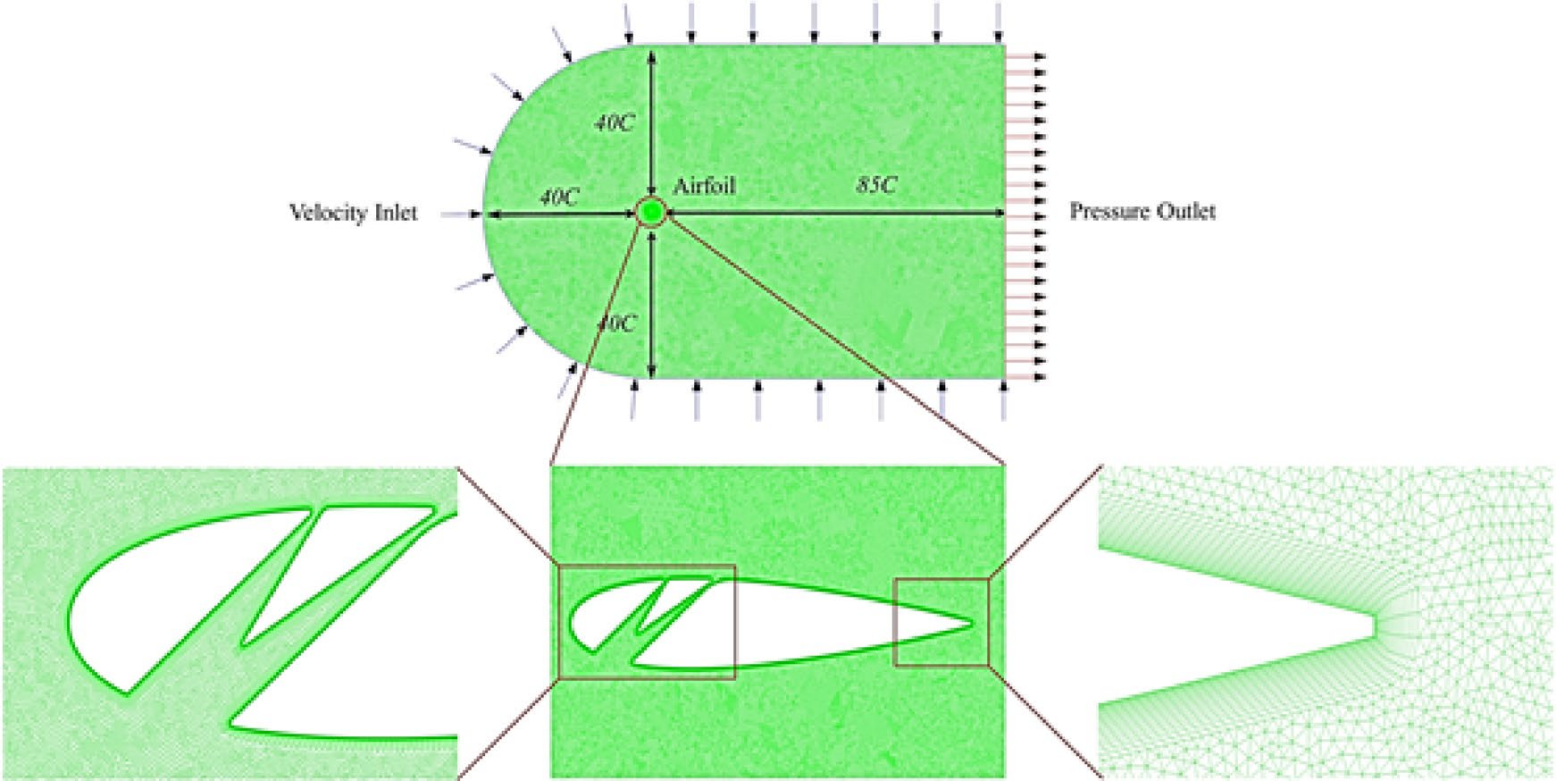
Meshing of the computational domain (top), around the leading edge (bottom left), the complete airfoil (bottom mid) and trailing edge (bottom right) of the double-split slotted airfoil.

A two-dimensional analysis is sufficient to predict the flow physics induced by the considered geometrical parameters. Thus, the simulation of an isolated static airfoil adequately meets the objectives of this study. Previous research has shown that two-dimensional flow field simulations of isolated static airfoils provide a reliable and efficient method for accurately predicting flow physics^25,73^. A C-grid domain was selected for its versatility in handling variations in the incoming flow direction along the semi-circular inlet boundary, with dimensions matching those (Fig. 3) used by Mohamed et al.^45^.

A pressure-based steady-state approach was employed, assuming incompressible air with a constant density of *ρ* = 1.225 kg/m^3^ and a dynamic viscosity of μ = 1.7894 kg/m−^−S^. The simulations were conducted at a chordwise Reynolds number of *R*_*e*_ = 250,000, representing a low turbulence regime^69^, with a freestream velocity of U_∞_ = 7.769 m/s and an outlet pressure of 1 atm. The airfoil’s wall was kept as a no-slip stationary wall. A coupled scheme algorithm was utilised to link pressure and velocity terms. The least squares cell-based method was applied for mesh adaptability, and a second-order upwind scheme minimised interpolation errors in convection terms (Ahmad et al. n.d.;^72^). The simulation was run until the residual convergence criterion dropped below 10^−5^. It was then continued to confirm stable *C*_*L*_ and *C*_*D*_ values without fluctuations.

## Results and discussion

### Mesh topology and grids

**Fig. 4:**
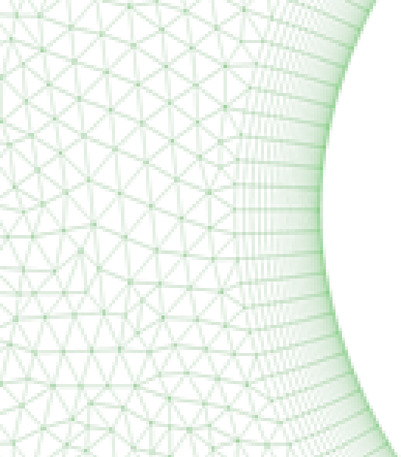
Inflation layers on the leading edge of the airfoil.

**Fig. 5:**
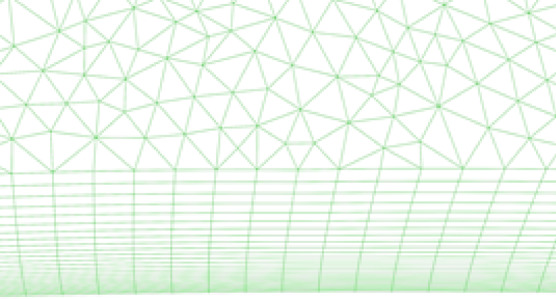
Inflation layers on the surface of the airfoil.

In the near-wall region of the airfoil, 25 quadrilateral cells were employed within the inflation layers (Figs. 4 and 5) at a growth rate of 1.1 to capture boundary layer phenomena accurately. The rest of the computational domain consisted of unstructured triangular cells. A Y + value of approximately 1 was maintained to enable the application of enhanced wall treatment. Mesh skewness was controlled below 0.6, with the majority of cells exhibiting orthogonality values between 0.6 and 0.9.

A grid independence test (GIT) was conducted to assess the impact of cell size on the solution accuracy by various grid densities. The test aimed to ensure accurate results while minimising mesh density. The evaluation focused on three key parameters: *C*_*L*_*, C*_*D*_ and* C*_*L*_*/C*_*D*_.

**Fig. 6:**
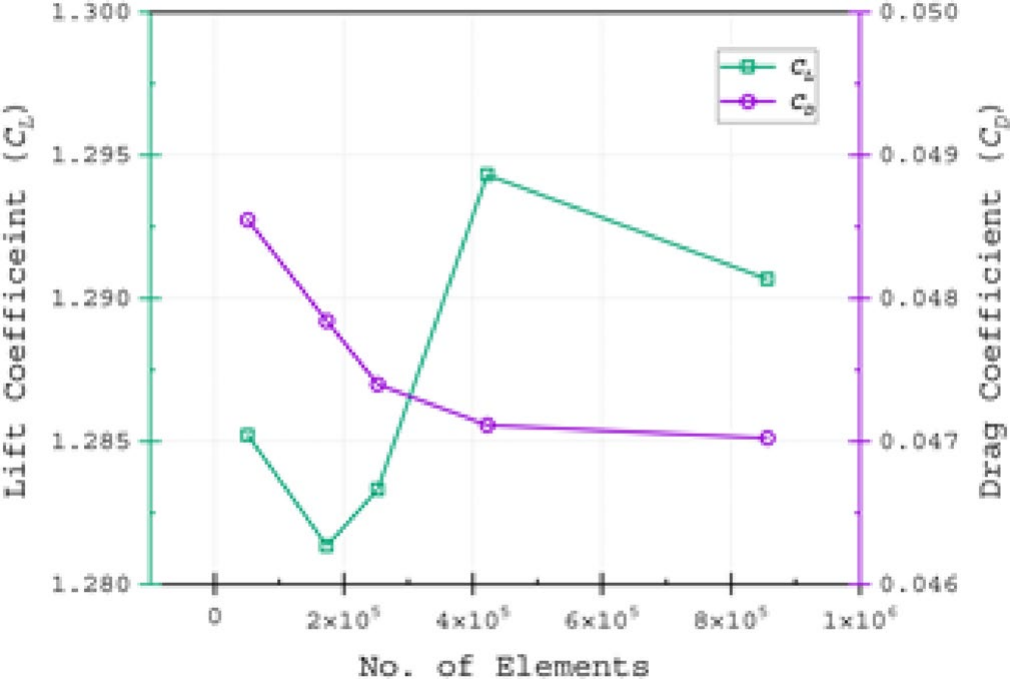
Variation of *C*_*L*_ and *C*_*D*_ against the number of elements for different mesh topology at *α* = 15°

These aerodynamic coefficients were evaluated at AOA of *α* = 15° for four different tests run with different mesh arrangements. At this particular AOA the separation, stall, and other complex flow events appeared around the airfoil. The solution achieved grid independence to the results with the G3 case, as *C*_*D*_ values showed no significant variations, showcased in (Fig. 6 and Table 3). The *C*_*L*_ exhibited oscillatory convergence^74^, with variations of 0.9% and 0.6% for the G4 and G5 grids, respectively. Similarly, *C*_*D*_ variations were within 0.7% and 0.8% compared to the G3 grid topology (in Table 3). The glide ratio (*C*_*L*_*/C*_*D*_) differences were under 1.45% and 1.4% for G4 and G5 grids, respectively. The G4 grid topology, comprising approximately 422,000 elements, was selected for this study. It provided a balance between computational accuracy and efficiency while ensuring reliable results. This grid size enhanced precision without imposing excessive computational demands.

**Table 3:** Data of grid independence test.

Grid case	No. of elements	C_L_	C_D_	C_L_/C_D_
G1	51,387	1.285	0.0485	26.472
G2	174,003	1.281	0.0478	26.783
G3	252,544	1.283	0.0473	27.075
G4	422,334	1.294	0.0471	27.472
G5	856,622	1.290	0.0470	27.448

### Verification and accuracy of the present model

**Fig. 7:**
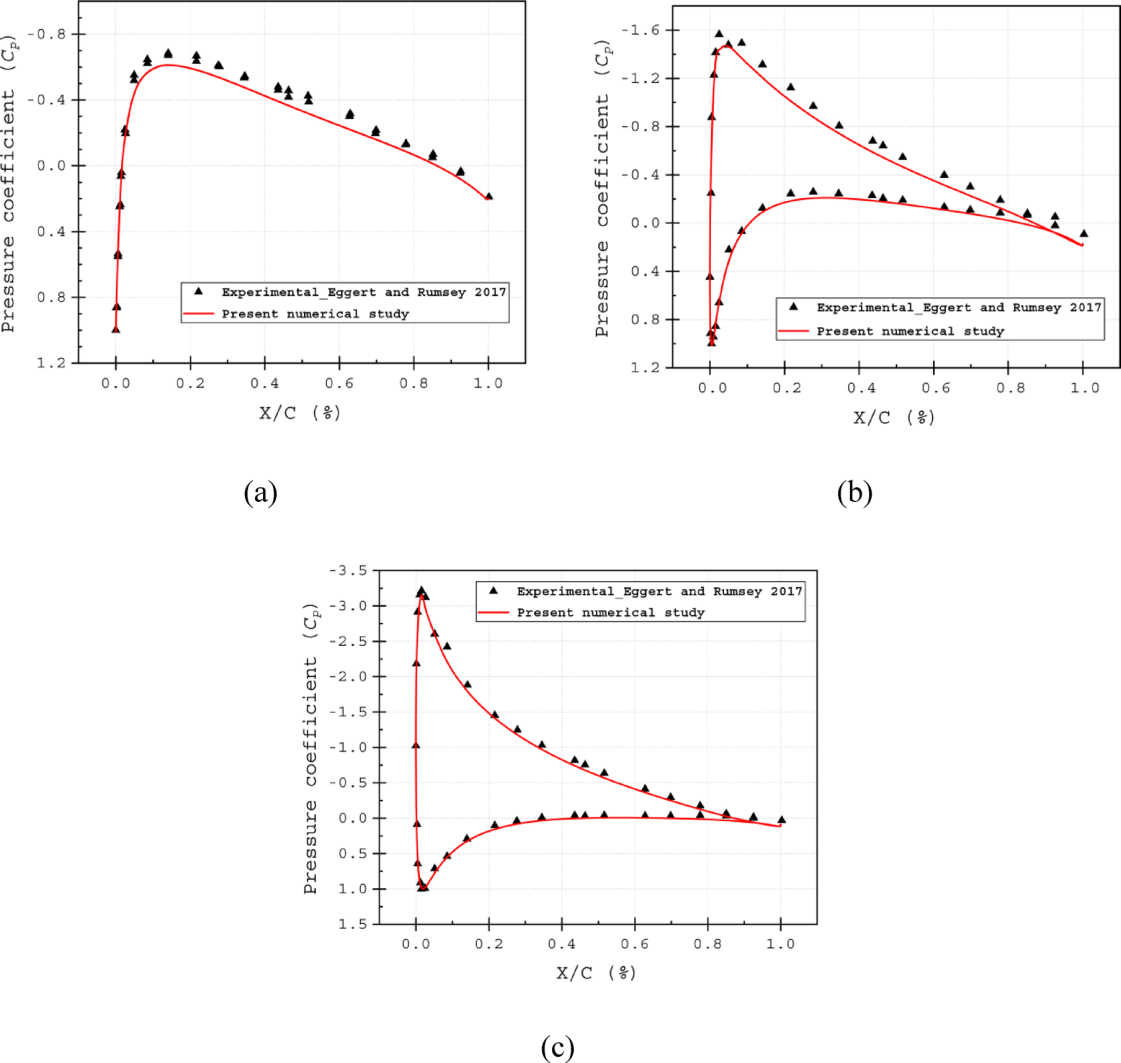
Pressure coefficient (*C*_*P*_) of Experimental data^69^ and present Computational result at (**a**) α = 0°, (**b**) α = 5° and (**c**) α = 10°

To validate the current numerical model, the pressure coefficient distribution along the normalised chord length was compared for various AOAs with experimental data from a prior study^69^. Figure 7a, b and c present the results of the pressure coefficient (*C*_*P*_) for the computational baseline airfoil and the experimental data. The comparisons show that the numerical simulations closely match the experimental trends, confirming the model’s accuracy. The pressure coefficient is defined as,

7 $${C_P} = \frac{{P - {P_\infty }}}{{\frac{1}{2}\rho V_\infty ^2}}$$

where *P* is the local static pressure across the blade surface, ${P}_{\infty }$ is the static pressure and the $\frac{1}{2}\rho {V}_{\infty }^{2}$ is the dynamic pressure of the free-stream air.

**Fig. 8:**
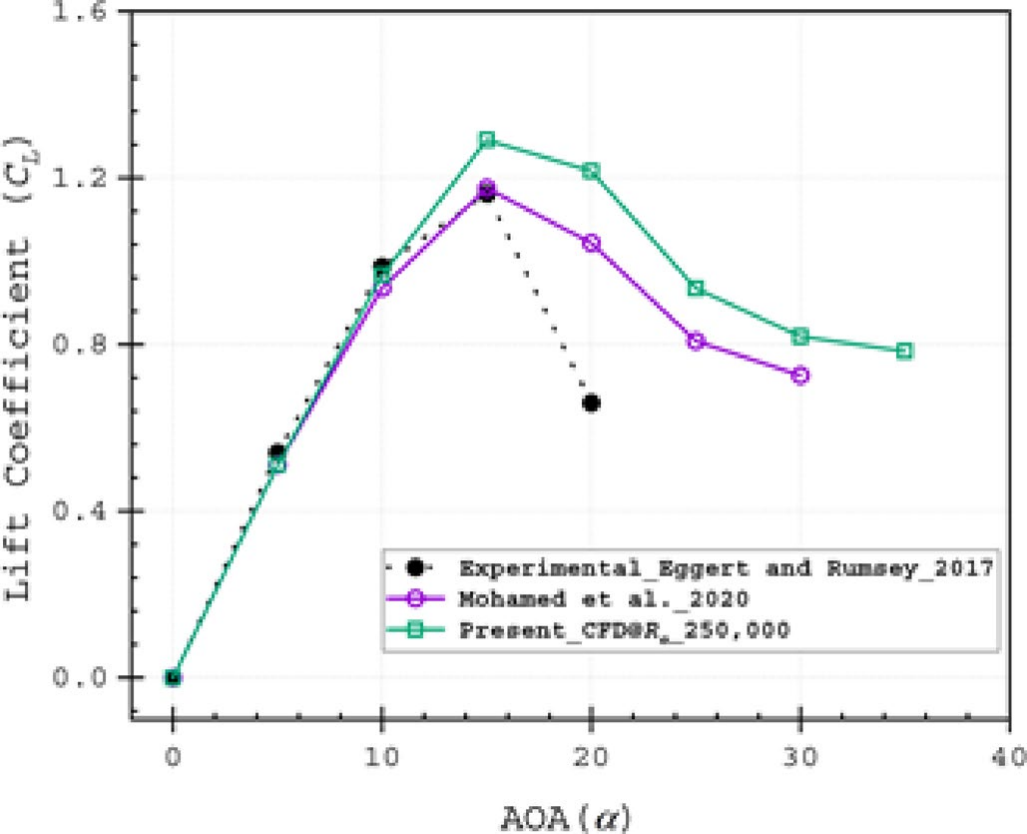
Lift coefficient (*C*_*L*_) of Experimental data^69^ and previously reported Numerical data^45^ and present Computational data.

In the absence of existing data for the double-split NACA 0018 airfoil, two-dimensional steady-state simulations were performed on a clean baseline NACA 0018 airfoil. To validate the numerical model, the computational results were compared with experimental data^69^ and computational data^45^. The comparisons showed similar patterns and flow physics, as illustrated in Fig. 8, confirming the model’s reliability.

The following section presents a detailed analysis of how individual geometric parameters affect the aerodynamic force coefficients *C*_*L*_*, C*_*D*_*, C*_*L*_*/C*_*D*_.

8 $${C}_{L}=\frac{{F}_{L}}{\frac{1}{2}\uprho {V}_{rel}^{2}{A}_{blade}}$$

9 $${C}_{D}=\frac{{F}_{D}}{\frac{1}{2}\uprho {V}_{rel}^{2}{A}_{blade}}$$

10 $$Lift-over-drag\,ceofficient=\frac{{C}_{L}}{{C}_{D}}$$

In Eqs. 8*F*_*L*_ and Eq. 9*F*_*D*_ are the lift force and drag force, respectively, where $\frac{1}{2}\rho {V}_{\infty }^{2}$ is the dynamic pressure for the free-stream air and ${A}_{blade}$ represents the area (chord length) of the airfoil surface.

### Effect of slot outlet location

The incorporation of a slot into a conventional airfoil significantly enhances aerodynamic performance. The narrow passage facilitates the ejection of high-momentum flow from the pressure surface to the suction surface, energising the boundary layer and effectively suppressing flow separation. This mechanism not only delays separation but also improves overall aerodynamic efficiency without introducing substantial drawbacks. The aerodynamic performance of an airfoil is characterised by key parameters such as the lift coefficient (*C*_*L*_), drag coefficient (*C*_*D*_) and and lift-to-drag ratio (*C*_*L*_*/C*_*D*_). Among these, *C*_*L*_ serves as a direct indicator of airfoil performance, reflecting its ability to generate lift under varying flow conditions.

The analysis focused on assessing the aerodynamic force coefficients of the Baseline and Slotted airfoils across α = 0◦−35◦. The aerodynamic performance of a double-split slotted airfoil is strongly sensitive to the slot outlet locations. Therefore, this section investigates the influence of the outlet position parameter by systematically varying its position. Six slot configurations were derived from the baseline airfoil by varying the suction-side slot midline location (B), designated as Type 1 slots see (Table 2). The B locations were positioned at 20%, 30%, 40%, 50%, 60%, and 70% of the chord length (denoted as 20C, 30C, 40C, 50C, 60C, and 70C, respectively). The pressure-side centreline location (A), outlet width (W2), and Y value remained constant. As B shifted toward the trailing edge (TE), the inlet width (W1) increased, allowing the slot to capture the stagnation point at higher angles of attack (AOAs).

**Fig. 9:**
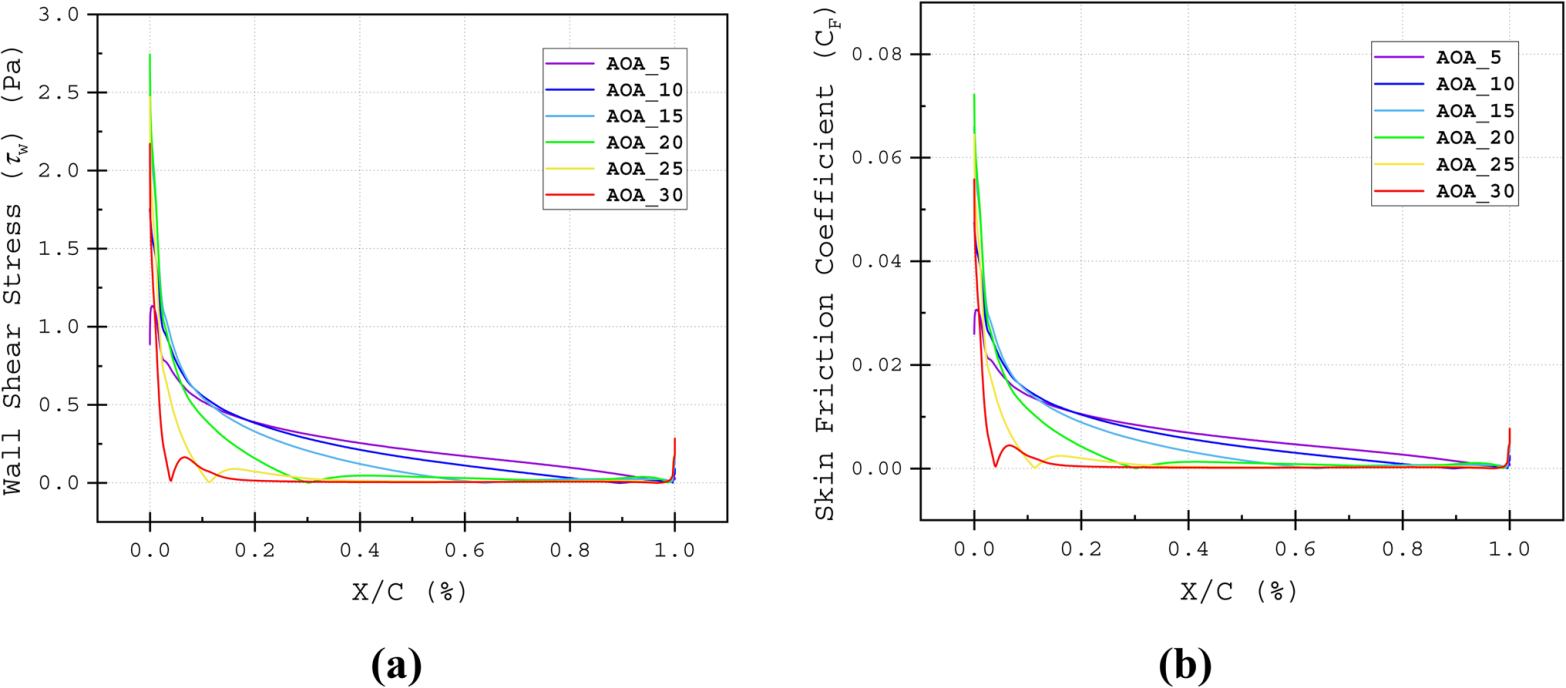
(**a**) Wall Shear Stress and (**b**) Skin Friction Coefficient against nondimensional chord length (*X/C*) for the upper surface of the baseline airfoil at various angles of attacks, α = 0°, α = 5°, α = 10°, α = 15°, α = 20°, α = 25°, α = 30°.

The stagnation points, illustrated in Fig. 20a–f, were analysed using the *C*_*P*_ distribution of the baseline airfoil. Additionally, Fig. 9a and b highlights the separation points on the suction surface at different AOAs. This study aims to determine the optimal slot outlet location for enhanced aerodynamic performance.

**Fig. 10:**
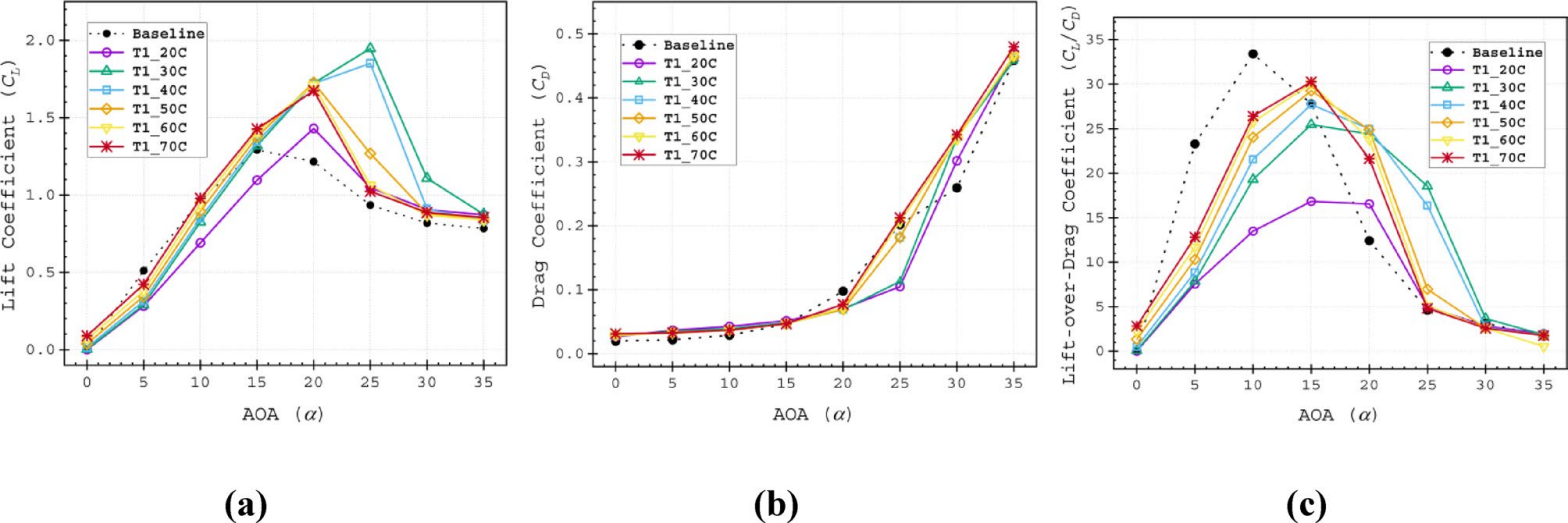
Aerodynamic (**a**) lift coefficient (*C*_*L*_), (**b**) lift coefficient (*C*_*D*_), (**c**) lift-over-drag coefficient (*C*_*L*_*/C*_*D*_) of Type 1 slot configurations.

The *C*_*L*_ for both baseline airfoil and slotted airfoils increases with the AOA. However, as shown in Fig. 10a–c, the baseline airfoil underperforms compared to the slotted configurations at low AOAs (0° ≤ α ≤ 10°). This reduction in lift is attributed to flow disturbances induced by the slot in the airfoil profile. Beyond *α* = 15°, all slotted configurations except for the T1_20*C* exceed the baseline *C*_*L*_. At higher AOA (17.5° ≤ *α* ≤ 35°), all slots variants maintain higher *C*_*L*_ values, with T1_30C achieving a peak *C*_*L*_ of 1.947 at α = 25°, which is more than double the baseline *C*_*L*_. Notably, T1_30C and T1_40C stalled at α = 25°, while the remaining configurations stalled earlier at α = 20°. Placing the slot outlet immediately upstream of the separation point maximises energy transfer to the boundary layer, as the high-energy fluid ejected from the slot effectively suppresses flow separation, thereby enhancing aerodynamic performance. This observation aligns with the findings of Bhavsar et al.^43^. Conversely, if the slot outlet is positioned too far upstream of the separation point, the injected fluid dissipates excess energy before reaching the critical separation zone, leading to lowered performance.

The presence of the slot induced drag, which is prominent below α = 15°, as all Type 1 slots generated higher drag coefficients *C*_*D*_ than the baseline. However, within the range of 15° ≤ α ≤ 27.5°, slots demonstrated lower *C*_*D*_ values compared to the baseline. At α = 25°, only T1_60C and T1_70C showed elevated *C*_*D*_. Additionally, drag increased as the slot outlet positions moved closer to the TE.

All Type 1 slot configurations exhibited a lower peak of *C*_*L*_*/C*_*D*_ curve compared to the baseline airfoil; however, they performed better over a wider AOA range shown in Fig. 10c. The analysis revealed that slot outlets positioned closer to the LE performed more effectively over a wide AOA range, with a lower *C*_*L*_*/C*_*D*_ peak. Contrarily, slots closer to the TE achieved higher peak *C*_*L*_*/C*_*D*_ values but within a narrower operational range. Higher *C*_*L*_*/C*_*D*_ values over a wider AOA range are beneficial for improving slotted airfoil performance.

The objective of this study is to minimise both the increase in drag coefficient (*C*_*D*_) and the reduction in lift coefficient (*C*_*L*_). Results demonstrate that as the angle of attack increases, both *C*_*D*_ and *C*_*L*_ exhibit an increasing trend. Therefore, the lift-to-drag ratio (*C*_*L*_*/C*_*D*_) provides a more suitable evaluation metric for optimal slot placement compared to analysing *C*_*L*_ and *C*_*D*_ separately^41^. All Type 1 slot configurations showed lower peak *C*_*L*_*/C*_*D*_ values than the baseline airfoil, but they maintained better performance across a wider range of angles of attack (AOAs), as shown in Fig. 10c. The analysis indicated that slot outlets positioned closer to the leading edge (LE) performed more effectively over a broad AOA range, though with a reduced peak *C*_*L*_*/C*_*D*_. In contrast, slots near the trailing edge (TE) achieved higher peak *C*_*L*_*/C*_*D*_ values but operated effectively only within a narrower AOA range.

**Fig. 11:**
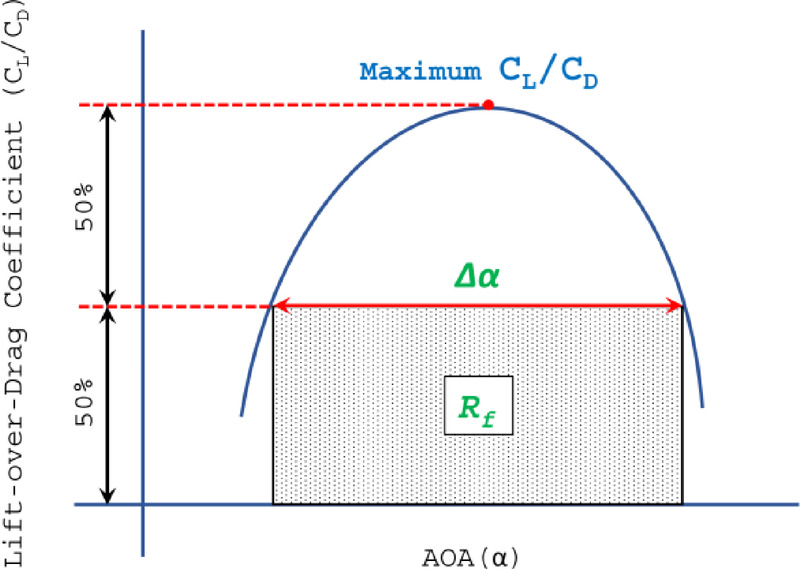
Detailed illustration of the Range factor (*R*_*f*_) used to evaluate the performance of slotted airfoils.

Since maximising (*C*_*L*_*/C*_*D*_) across a wide operational range is crucial for slotted airfoil performance, these findings underscore key trade-offs in slot positioning. To evaluate optimal slotted airfoil designs using a single comprehensive metric,^45^ introduced a novel technique based on two key parameters: half of the maximum (*C*_*L*_*/C*_*D*_)_max and the effective angle-of-attack range (Δα) within which this half-peak performance is sustained (Fig. 11). These parameters are combined into a unified measure, the Range factor (Rf), which quantifies an airfoil’s ability to maintain near-optimal performance over an extended operational range. This metric provides a critical basis for determining the most efficient slotted airfoil configuration. The Range factor (*R*_*f*_) is defined as,

11 $$Rf=\left[\frac{{{(C}_{L}/{C}_{D})}_{max}}{2}\right]\Delta \alpha$$

**Fig. 12:**
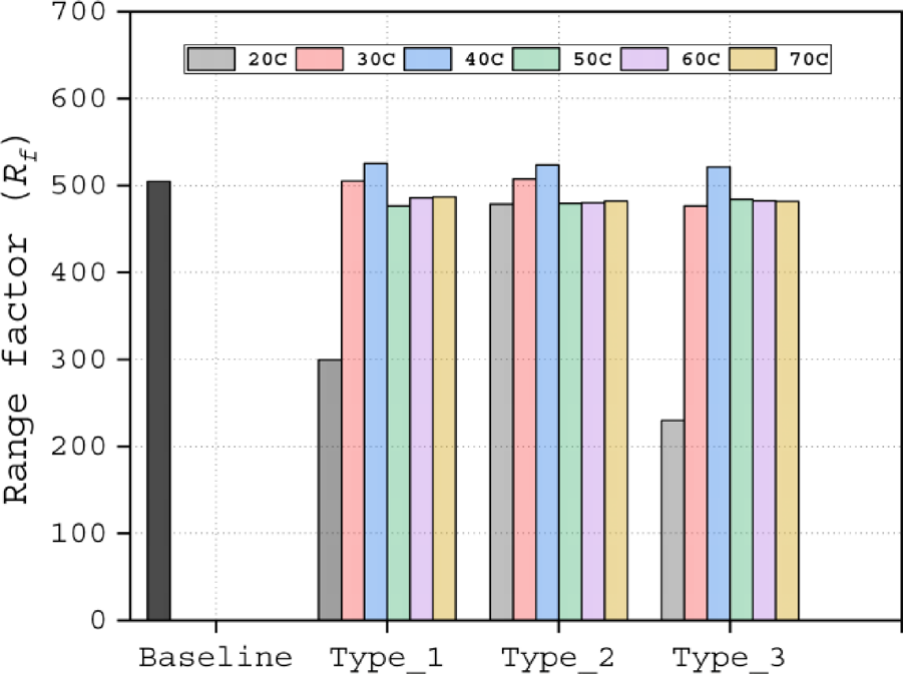
Range factor data of Type 1, Type 2 and Type 3 slot configurations.

Among the Type 1 slots, the T1_70C configuration achieved the highest value of *C*_*L*_*/C*_*D*_ at α = 15°, but exhibited the narrowest performance range. In contrast, the T1_30C slot demonstrated the widest *C*_*L*_*/C*_*D*_ profile (Fig. 10c). The T1_40C slot provided a balanced performance, delivering higher *C*_*L*_*/C*_*D*_ than the T1_30C while maintaining a broader operational range compared to the T1_70C. The Range factor (*R*_*f*_) further validated the superiority of the T1_40C configuration, yielding the highest value of 525.4 (Table 4, Fig. 12), confirming its optimal design. Aerodynamic force analysis demonstrates that the slot outlet location critically influences aerodynamic performance.

**Table 4:** Range factor (*R*_*f*_) for Type 1, Type 2 and Type 3 slot configurations.

Baseline airfoil	504.5					
Slotted airfoils	20C	30C	40C	50C	60C	70C
Type 1	299.7	505.0	525.4	476.6	485.8	486.9
Type 2	478.4	507.4	523.5	479.2	480.3	482.2
Type 3	229.7	476.6	521.2	484.1	482.5	481.7

**Fig. 13:**
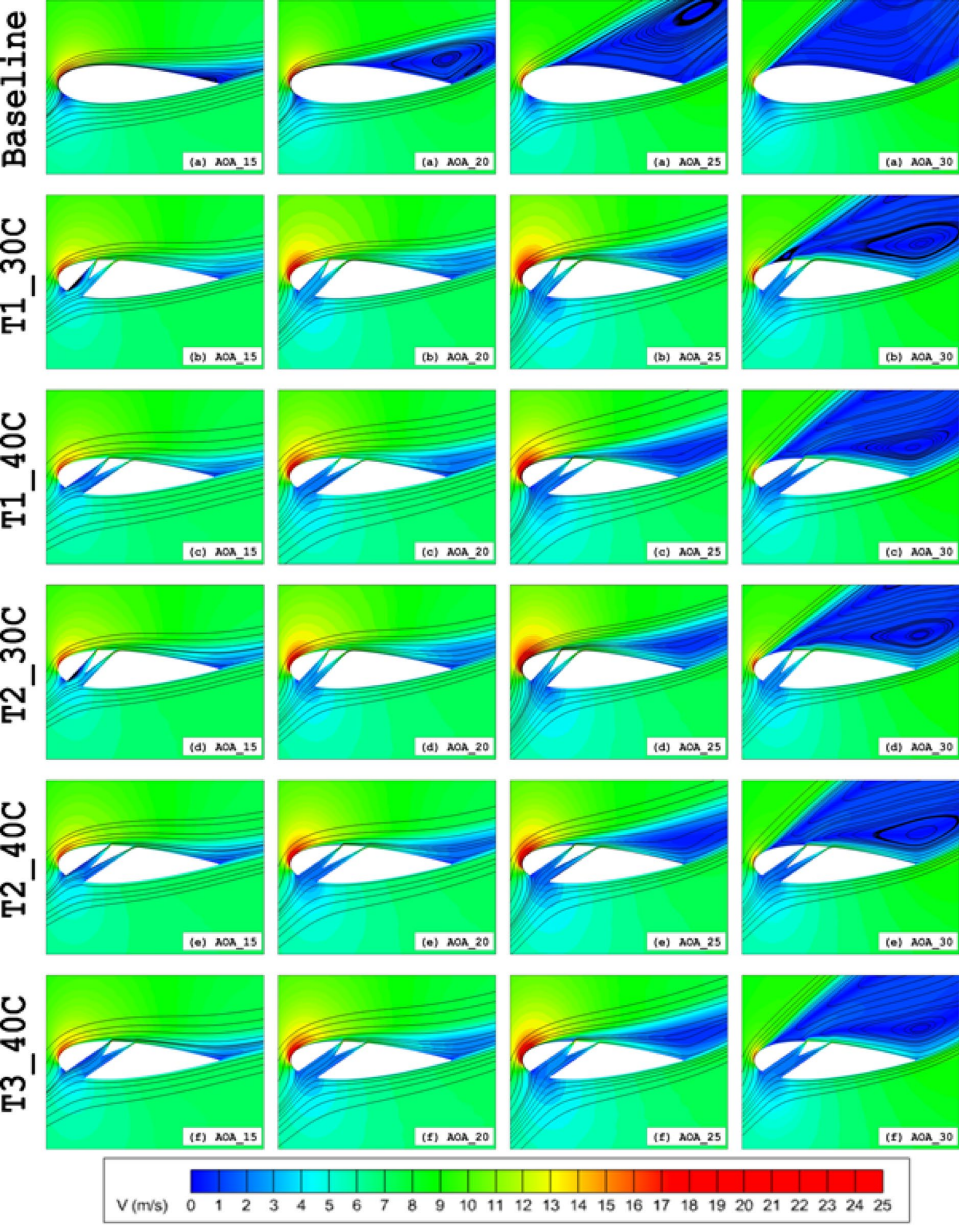
Velocity field contours superimposed with velocity streamlines for Baseline airfoil and various slot configurations.

Flow visualisation in Fig. 13 suggested, for the baseline configuration, stall took place at *α* = 15°, significant flow separation occurred at α = 20° (Fig. 8a), whereas all Type 1 slot cases maintained attached flow. At α = 25°, the T1_30C and T1_40C slots continued to effectively delay separation, resulting in higher CL values, which are satisfied by the force analysis (Fig. 10a) and flow contours (Fig. 13).

Key findings include:

#### Outlet position effect

A leading-edge (LE) outlet enhances performance at high angles of attack α ≥ 20∘, while a trailing-edge (TE) outlet is more effective at low angles α ≤ 15°. At low α, separation initiates near the TE, favouring TE outlets. Conversely, at high α, separation shifts toward the LE, making LE outlets more advantageous. This behaviour stems from the slot’s hollow passage linking the stagnation and separation points. The injected high-momentum flow re-energises the boundary layer through enhanced energy mixing, thereby delaying separation.

#### Slot inlet optimization

The inlet width must be carefully tuned—excessive width leads to kinetic energy losses within the slot channel. Since stagnation points on the pressure side remain confined to the 0C–15C region (up to α = 35°), an overly wide inlet is unnecessary and detrimental to performance.

#### Flow alignment and geometry effects

Proper alignment of flow direction with the slot’s split passages ensures efficiency. Misalignment causes flow impingement on the airfoil wall, increasing pressure, inducing vortices, and dissipating energy (Fig. 15). The sharp corner of the first airfoil element disrupts flow; introducing a filleted edge improves performance by smoothing flow redirection. While slotted airfoils can enhance aerodynamic performance under optimised conditions, improper slot design may degrade performance compared to the baseline configuration.

### Effect of the wedge (2nd element) length

The influence of wedge length was systematically evaluated for the six previously analysed slot configurations (in Section "Effect of slot outlet location") across identical angles of attack. The Type 1 slot configurations were further analysed under two distinct wedge tip height conditions (denoted as Y), measured from the airfoil’s bottom maximum thickness: Type 2 with Y = 7C and Type 3 with Y = 11C, (Refer to Fig. 2 and Table 2 for geometric details). A reduction in Y inherently increases both the wedge length (i.e., the airfoil’s second element) and the split channel length. This analysis aimed to characterize the effects of slot geometry and split channel length variations and identify the optimal wedge height configuration for maximizing slotted airfoil performance.

**Fig. 14:**
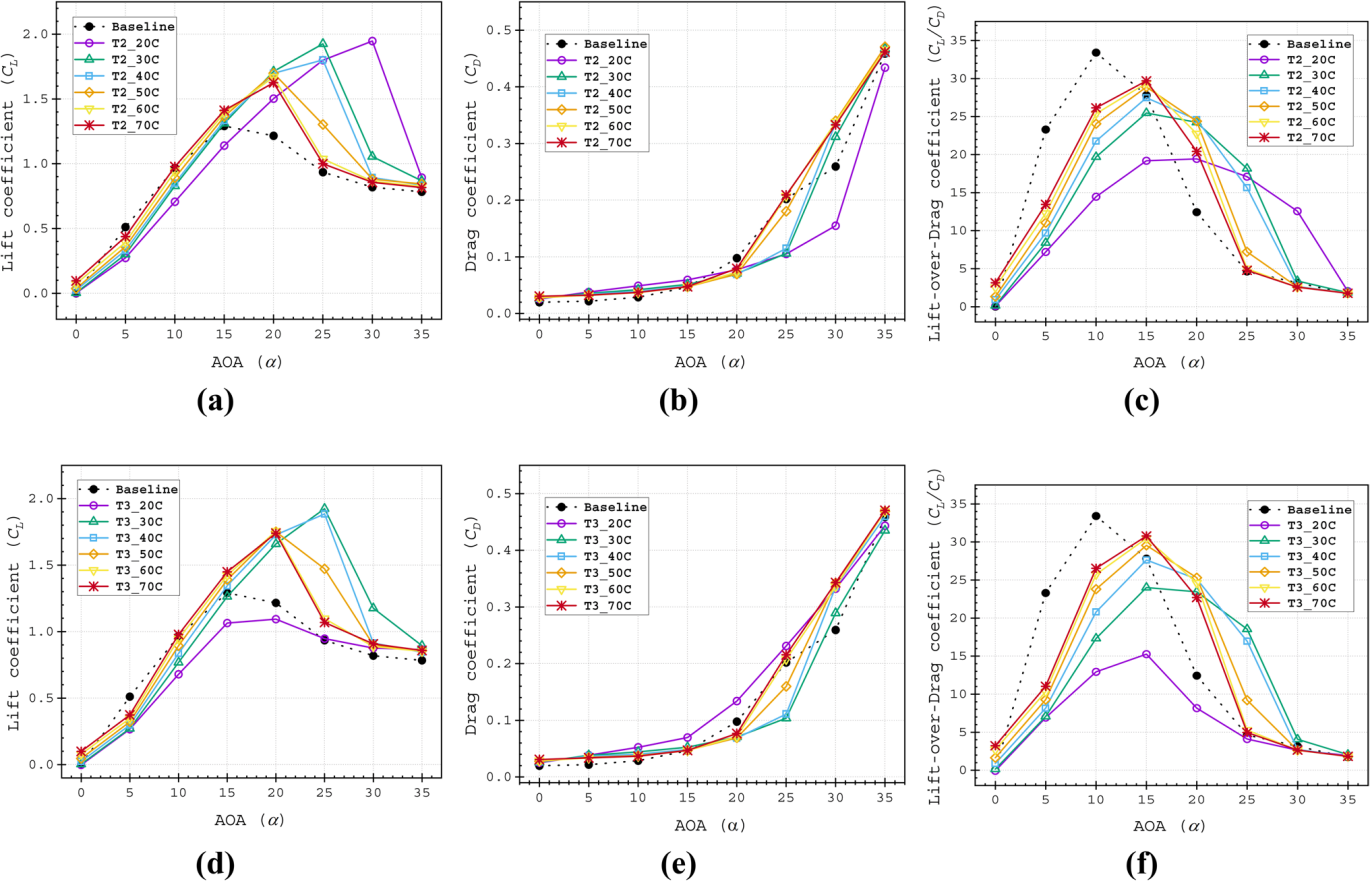
Aerodynamic (**a** and **d**) lift coefficient (*C*_*L*_), (**b** and **e**) drag coefficient (*C*_*D*_), (**c** and **f**) lift-over-drag coefficient (*C*_*L*_*/C*_*D*_) of Type 2 (**a**–**c**) and Type 3 (**d**–**f**) slot configurations.

Generally, a similar pattern of the *C*_*L*_ is observed for Type 1, Type 2 and Type 3 based on the slot outlet position. The T2_30C, T2_40C, T3_30C, and T3_40C slots stalled at α = 25°. The T2_50C, T2_60C, T2_70C, T3_50C, T3_60C, and T3_70C slots stalled at α = 20° Fig. 14a and d. However, the effect of the convergent split passage highlights that the shorter channels help to gain more performance for those that have having outlet near the TE at higher AOAs. The T2_30C, T2_40C, T3_30C, and T3_40C slots recorded peak *C*_*L*_ values of 1.925, 1.801, 1.924 and 1.884 at an AOA of α = 25°, respectively, consistent with velocity contour results, which showed smaller separation zones in Fig. 13. Notably, the T2_20C slot achieved the highest *C*_*L*_ of 1.946 at an AOA of α = 30°.

After stalling, Type 3 slots exhibited higher *C*_*D*_ than Type 2 for the corresponding slot configurations, Fig. 14b and e. The *C*_*D*_ distribution indicates that T2_20C had the lowest *C*_*D*_ at α = 30°, while T2_30C and T2_40C showed nearly identical *C*_*D*_ values at α = 25°, both lower than the baseline value Fig. 14b. The *C*_*L*_/*C*_*D*_ revealed that T2_20C had the broadest range but the lowest peak, while T2_30C, T2_40C, T3_30C and T3_40C slots demonstrated more balanced performance, satisfying the importance of the glide ratio to evaluate the optimal slot arrangement. The remaining slot configurations showed relatively poor performance. In summary, no such exceptional pattern was observed other than the slight improvement in *C*_*L*_*/C*_*D*_ for the configuration with slot outlets beyond 40C at high AOA (α ≥ 20°).

The Range factor (*R*_*f*_) analysis in Fig. 12 shows that T2_20C produced a 60% higher value compared to T1_20C. Additionally, T2_30C and T2_40C exhibited higher values than the remaining Type 2 slots. For the Type 3 slot, only T3_40C outperformed the baseline airfoil. Consistently, the 30C and 40C cases from Type 1 and Type 2, as well as the 40C case from the Type 3 slot, ranked at the top of the Rf scale, as shown in Table 4.

The interactive effects of slot outlet position and wedge length on slotted airfoil performance were systematically investigated. Downstream shifting of the slot outlet increases the passage length, while wedge length variations were implemented as follows: Type 2 slots manually increased wedge length, and Type 3 slots reduced wedge length (due to shorter inherent geometry).

**Fig. 15:**
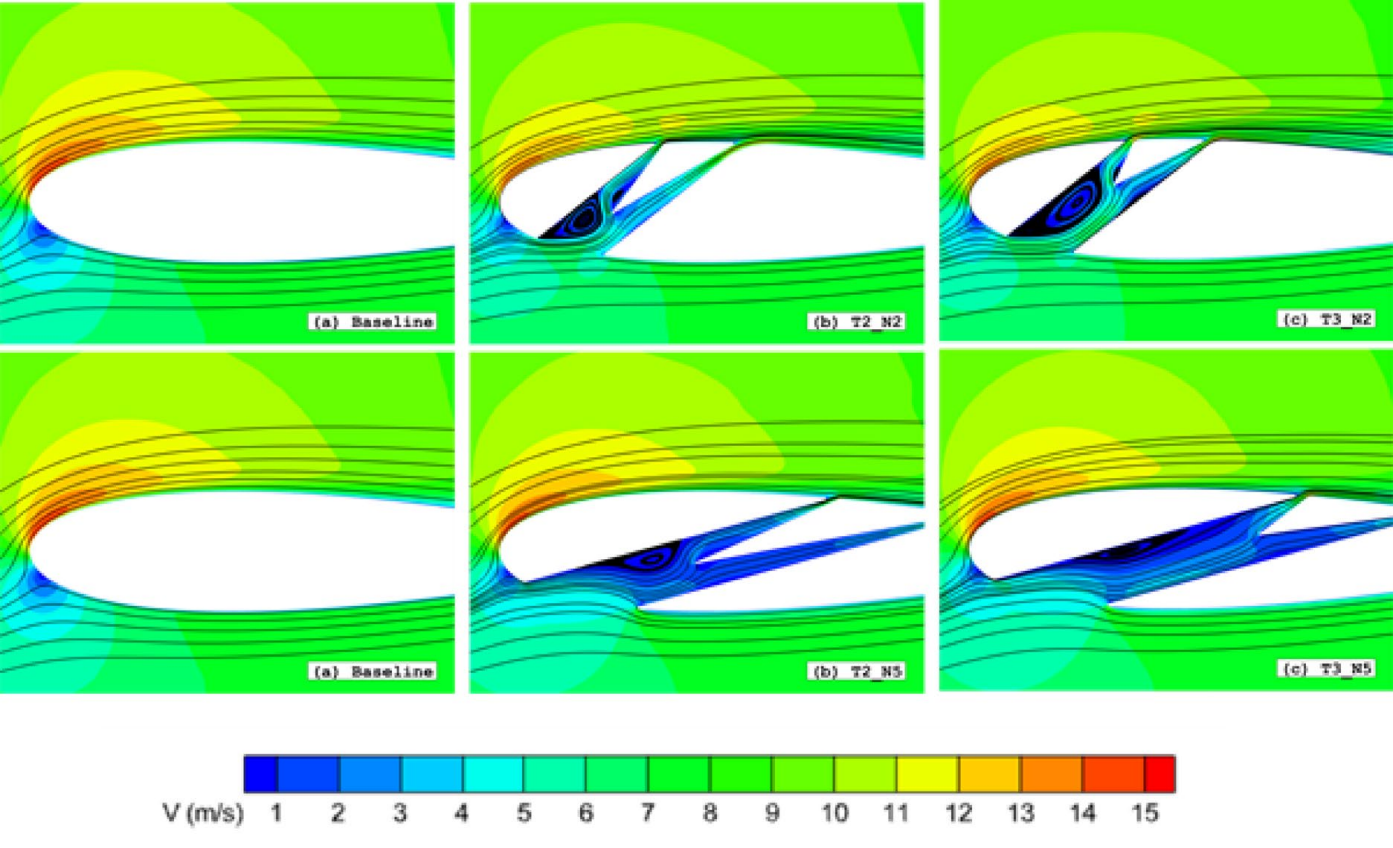
Velocity field contours superimposed with velocity streamlines for (**a**) Baseline, (**b**) Type 2 and (**c**) Type 3 slot configurations at α = 10°.

Key findings from this analysis reveal distinct performance trends across different angle-of-attack (AOA) regimes:

1. At low AOAs (0° ≤ α ≤ 15°), Type 2 configurations demonstrated superior performance compared to Type 3 slots. However, as the slot outlet approached the trailing edge (TE), Type 3 slots exhibited improved efficiency (Fig. 14a–f). This enhancement root from diminished vortex formation within the slot (Fig. 15), promoting more streamlined flow through the split channels.
2. At high AOAs (15° ≤ α ≤ 30°), Type 3 slots outperformed both Type 1 and Type 2 configurations with TE-proximate outlets. The performance improvement is attributed to mitigated flow separation due to reduced fluid-wall interactions within the wedge element, minimised kinetic energy losses and Enhanced flow rates through the slotted passage.

These results underscore the critical influence of flow characteristics through the first split channel on overall airfoil performance. Furthermore, the analysis demonstrates that wider slots near the leading edge (LE) are suboptimal for slotted blade architectures. Optimal performance requires co-optimisation of Wedge length (Y value) and Slot outlet location. These parameters exhibit strong interdependence, as confirmed by Range factor (Rf) analysis, which indicates superior performance of Type 1 and Type 2 slots under optimal conditions. Further investigation of slot configurations is presented in the following section.

### Effect of the Outlet Width of the Slot

This section evaluates the aerodynamic influence of slot outlet width variation for five optimal configurations (T1_30C, T1_40C, T2_30C, T2_40C, and T3_40C), selected from the initial 18 configurations analysed in Sections "Effect of slot outlet location" and "Effect of the wedge (2nd element) length" based on their Range factor (*R*_*f*_) performance metrics. The investigation maintained constant outlet widths (W2) for Type 1, 2, and 3 configurations, while systematically modifying W2 to 2C and 0.5C for Type 4 and Type 5 configurations, respectively. These geometric adjustments were implemented exclusively for the outlet width parameter, preserving all other airfoil dimensions unchanged. The Type 4 slot configurations were methodically derived from their parent configurations, with T4_T1_30C and T4_T1_40C originating from T1_30C and T1_40C, respectively, while T4_T2_30C and T4_T2_40C were developed from T2_30C and T2_40C. Similarly, T4_T3_40C was derived from the T3_40C baseline. Type 5 configurations followed an identical derivation methodology. Complete geometric specifications of these modified configurations are provided in Table 2.

Up to an AOA of *α* = 25°, T4_T1_30*C* and T4_T2_30*C* have demonstrated nearly identical performance because of their similar slot outlet location geometry at 30*C*. T4_T1_30*C* achieved the highest *C*_*L*_ of 2.0453, T4_T2_30*C* followed by a *C*_*L*_ of 2.0438 at *α* = 25°, while at *α* = 30°, T4_T2_30*C* excelled with a *C*_*L*_ of 1.9545.

**Fig. 16:**
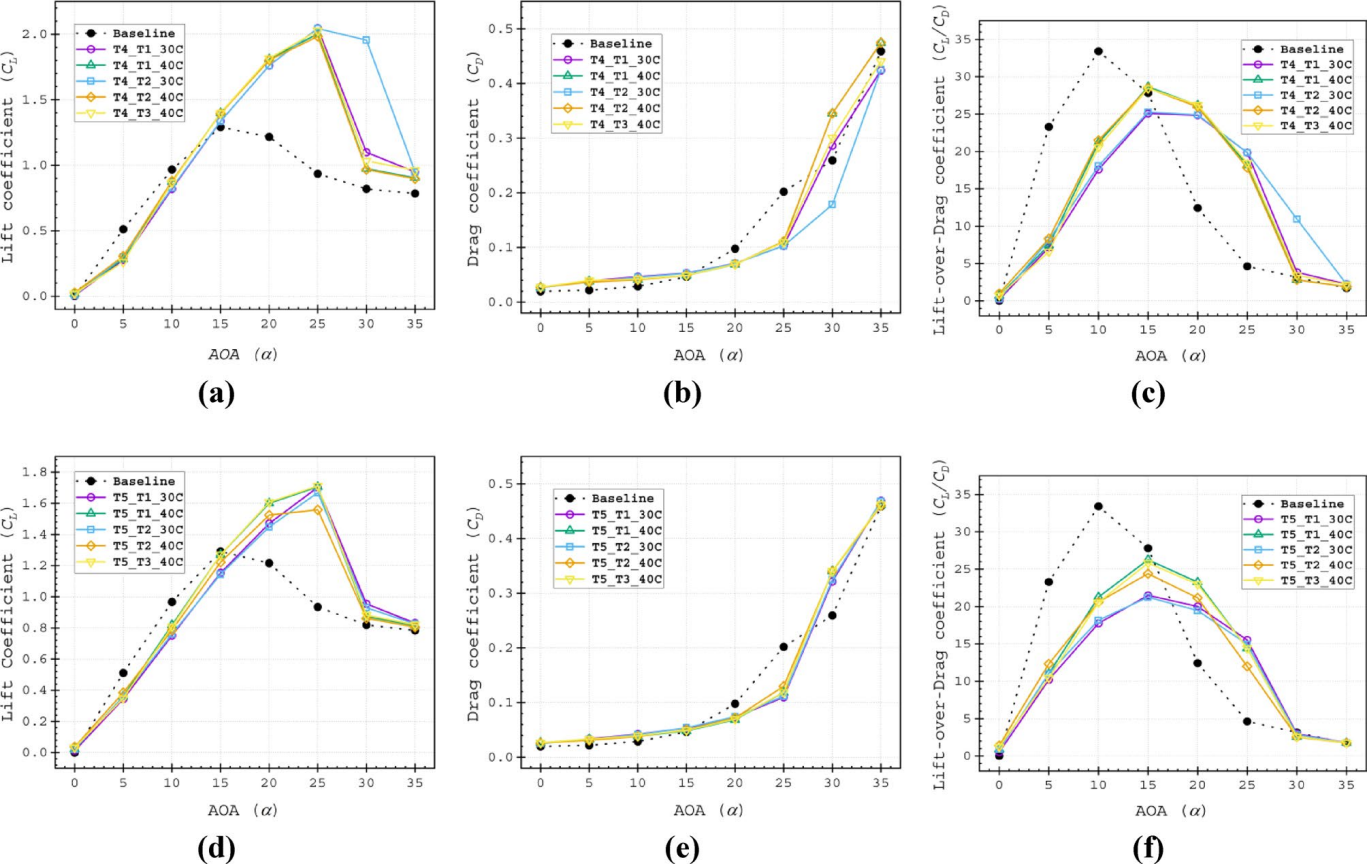
Aerodynamic (**a** and **d**) lift coefficient (*C*_*L*_), (**b** and **e**) drag coefficient (*C*_*D*_), (**c** and **f**) lift-over-drag coefficient (*C*_*L*_*/C*_*D*_) of Type 4 (**a**–**c**) and Type 5 (**d**–**f**) slot configurations.

Comparative analysis revealed consistent performance superiority of Type 4 slot configurations over Type 5 variants across all evaluated metrics (Fig. 16a–f). While the T4_T1_30C configuration achieved the greatest lift coefficient (*C*_*L*_) performance, its Range factor (*R*_*f*_) of 492.18 remained below the baseline value. In contrast, the four remaining Type 4 configurations demonstrated dual advantages: (1) exceeding baseline Rf performance and (2) achieving the highest overall scores among all tested slot configurations. Specifically, these exhibited Rf values of 536.63 (T4_T1_40C), 550.63 (T4_T2_30C), 539.70 (T4_T2_40C), and 531.46 (T4_T3_40C), as documented in Table 5. The Type 5 configurations consistently underperformed, representing the least effective slotted airfoil design approach in this study.

**Table 5:** Range factor (*R*_*f*_) for Type 4 and Type 5 slot configurations.

Baseline airfoil	504.5				
Slotted airfoils	T1_30C	T1_40C	T2_30C	T2_40C	T3_40C
Type 4	493.7	536.6	550.6	539.7	531.4
Type 5	463.2	512.4	467.3	486.7	501.3

**Fig. 17:**
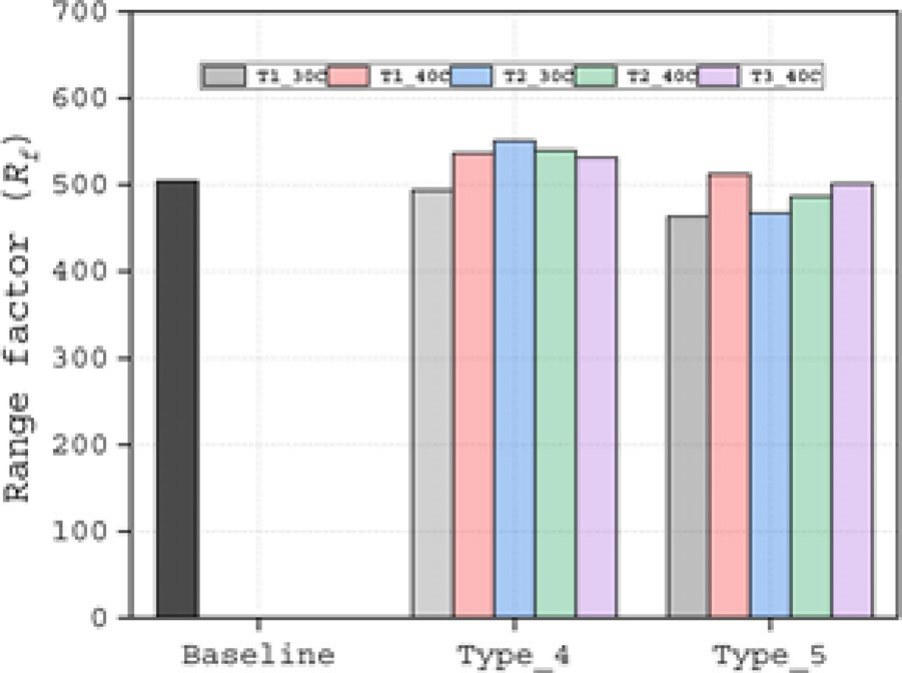
Range factor data of Type 4 and Type 5 slot configurations.

To complement the Range factor (*R*_*f*_) results, we present a comprehensive analysis of the key factors that substantiate the aerodynamic benefits of slot integration in airfoils. The range factor data of type 5 and type 5 slot configuration is depicted in Fig. 17. This investigation focuses exclusively on the T4_T2_30C configuration for three compelling reasons: (1) it demonstrated performance superiority, with an Rf value of 550.63 that significantly exceeds both baseline and alternative slot configurations; (2) its distinctive flow characteristics that set it apart from other slot designs; and (3) its geometric similarity to the baseline model, which facilitates direct performance comparison and unambiguous evaluation of slot-induced improvements.

### Analysis of optimal slot

**Fig. 18:**
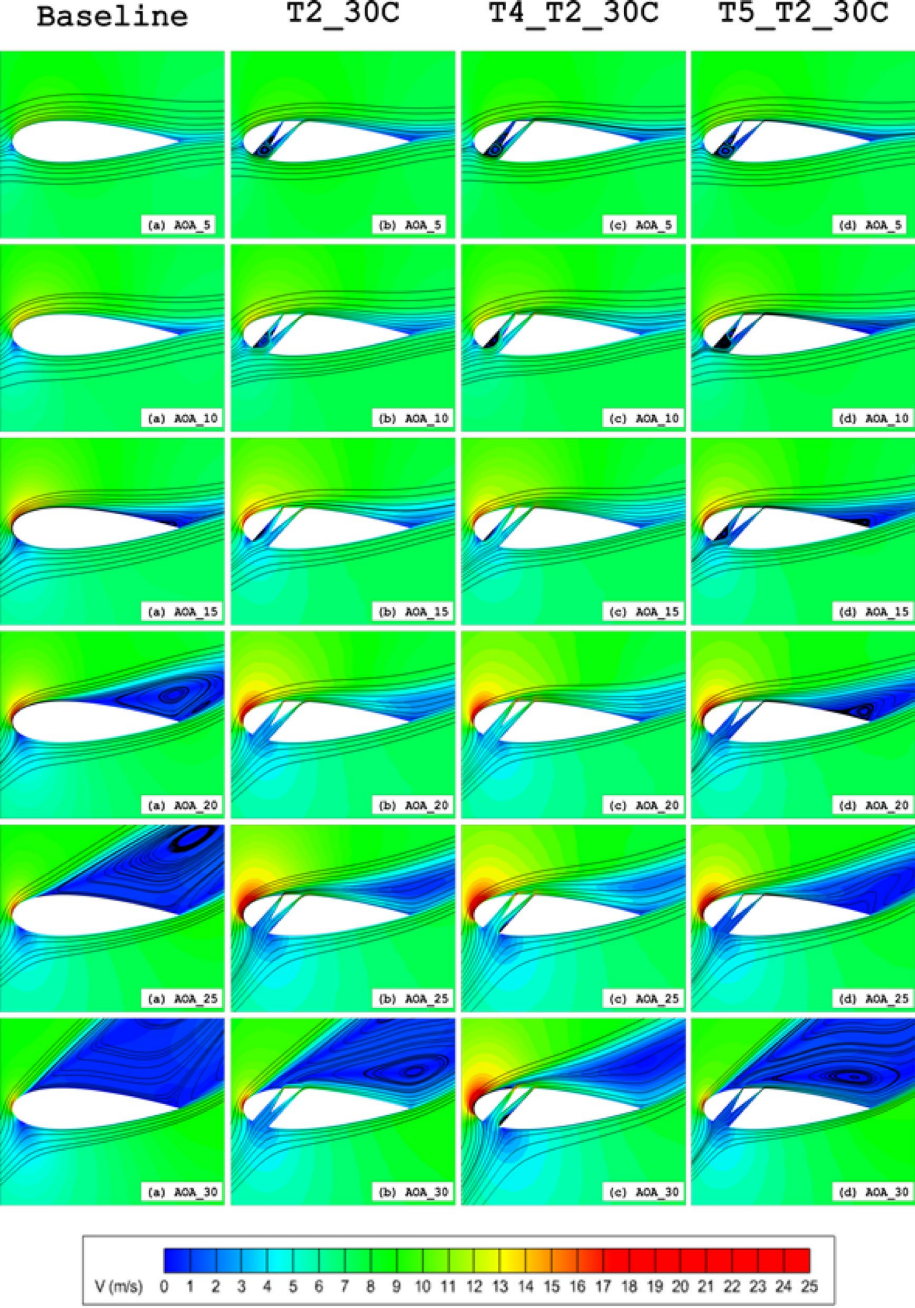
Velocity field contours superimposed with velocity streamlines for (**a**) Baseline, (**b**)T2_30*C*, (**c**) T4_T2_30*C* and (**d**) T5_T2_30*C* slot configurations at α = 5°, 10°, 15°, 20°, 25°, 30°

As the flow develops downstream along the airfoil surface with increasing angle of attack (AOA), the near-wall velocity profile decelerates, converting kinetic energy to pressure energy. This adverse pressure gradient promotes boundary layer separation and consequent drag augmentation. The slotted airfoil configuration mitigates this effect through boundary layer re-energisation, where the high-momentum slot outflow (Fig. 18) injects kinetic energy into the separating flow region. The resulting interaction between the slot jet and mainstream flow (Fig. 21a–h) produces two key effects: (1) enhanced surface flow acceleration, and (2) formation of a thinner, more stable boundary layer compared to the baseline configuration (highlighted in Fig. 21b–d), in terms of velocity gradients. This momentum transfer mechanism effectively suppresses the development of recirculation zones on the suction surface by maintaining attached flow throughout the operational AOA range.

**Fig. 19:**
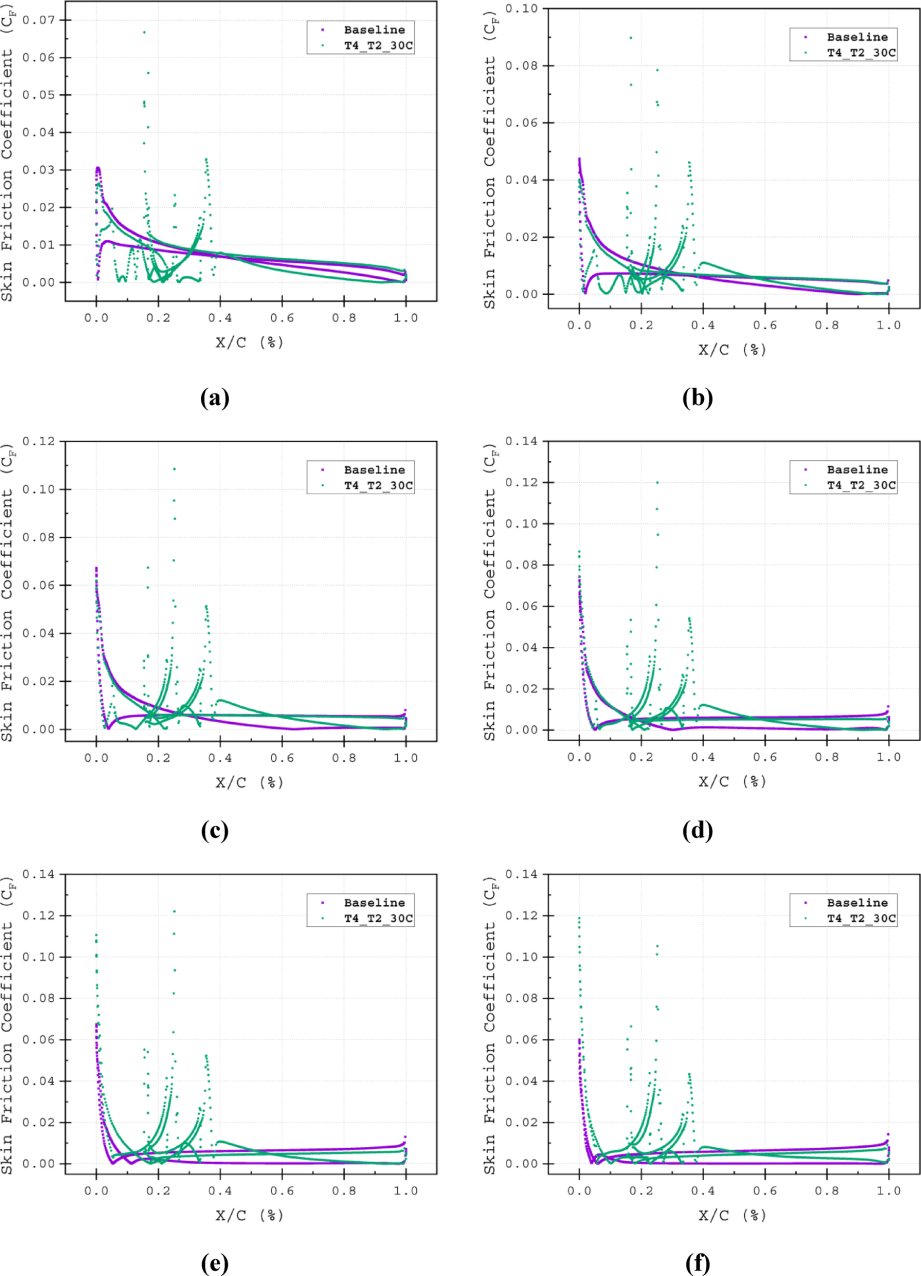
Skin Friction Coefficient (*C*_*F*_) of Baseline and T4_T2_30*C* slot configuration illustrated at (**a**) α = 5°, (**a**) α = 5°, (**b**) α = 10°, (**c**) α = 15°, (**d**) α = 20°, (**e**) α = 25° and (**e**) α = 30°.

**Fig. 20:**
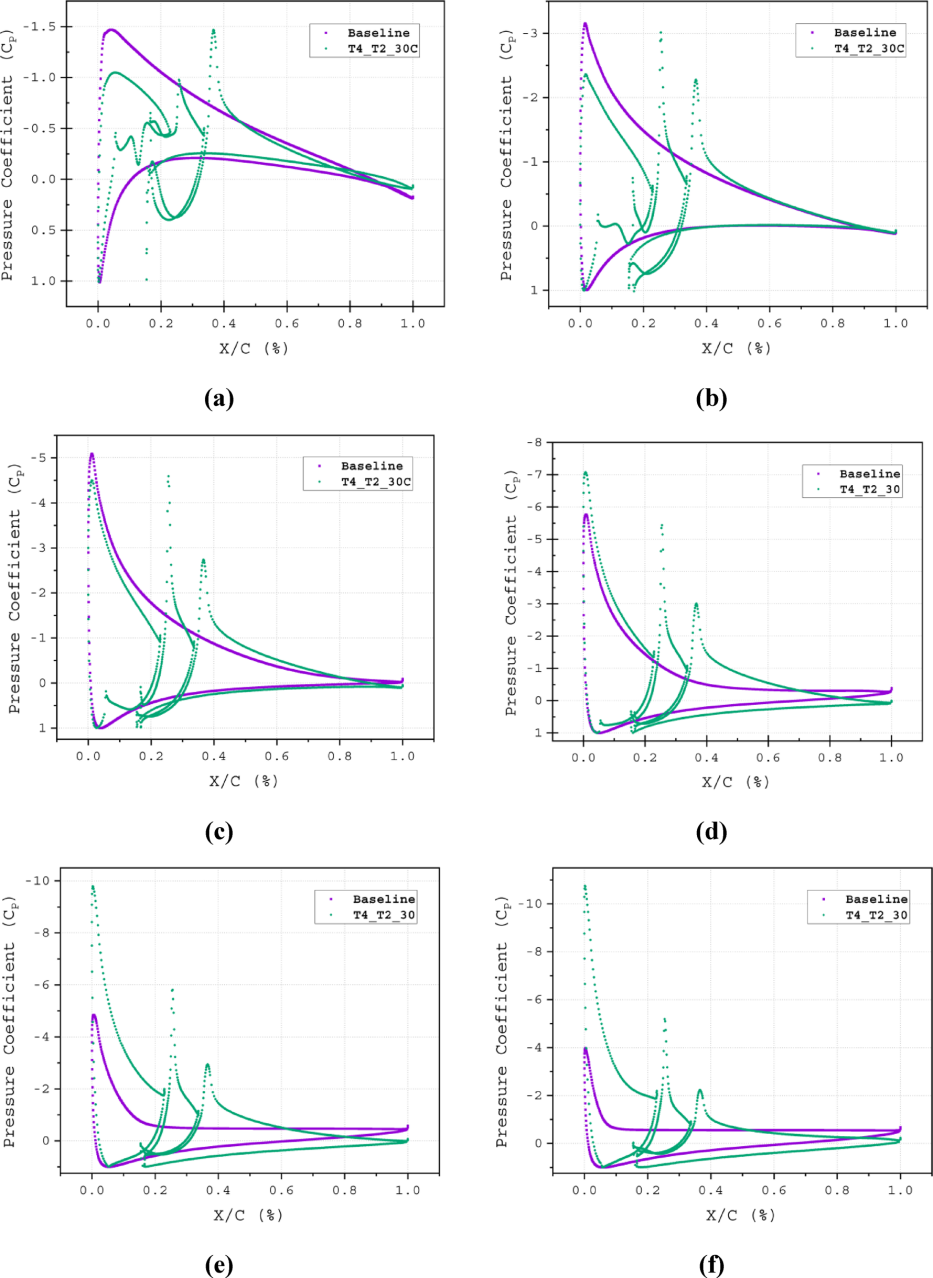
Pressure Coefficient (*C*_*P*_) of Baseline and T4_T2_30*C* slot configuration illustrated at (**a**) α = 5°, (**a**) α = 5°, (**b**) α = 10°, (**c**) α = 15°, (**d**) α = 20°, (**e**) α = 25° and (**e**) α = 30°.

**Fig. 21:**
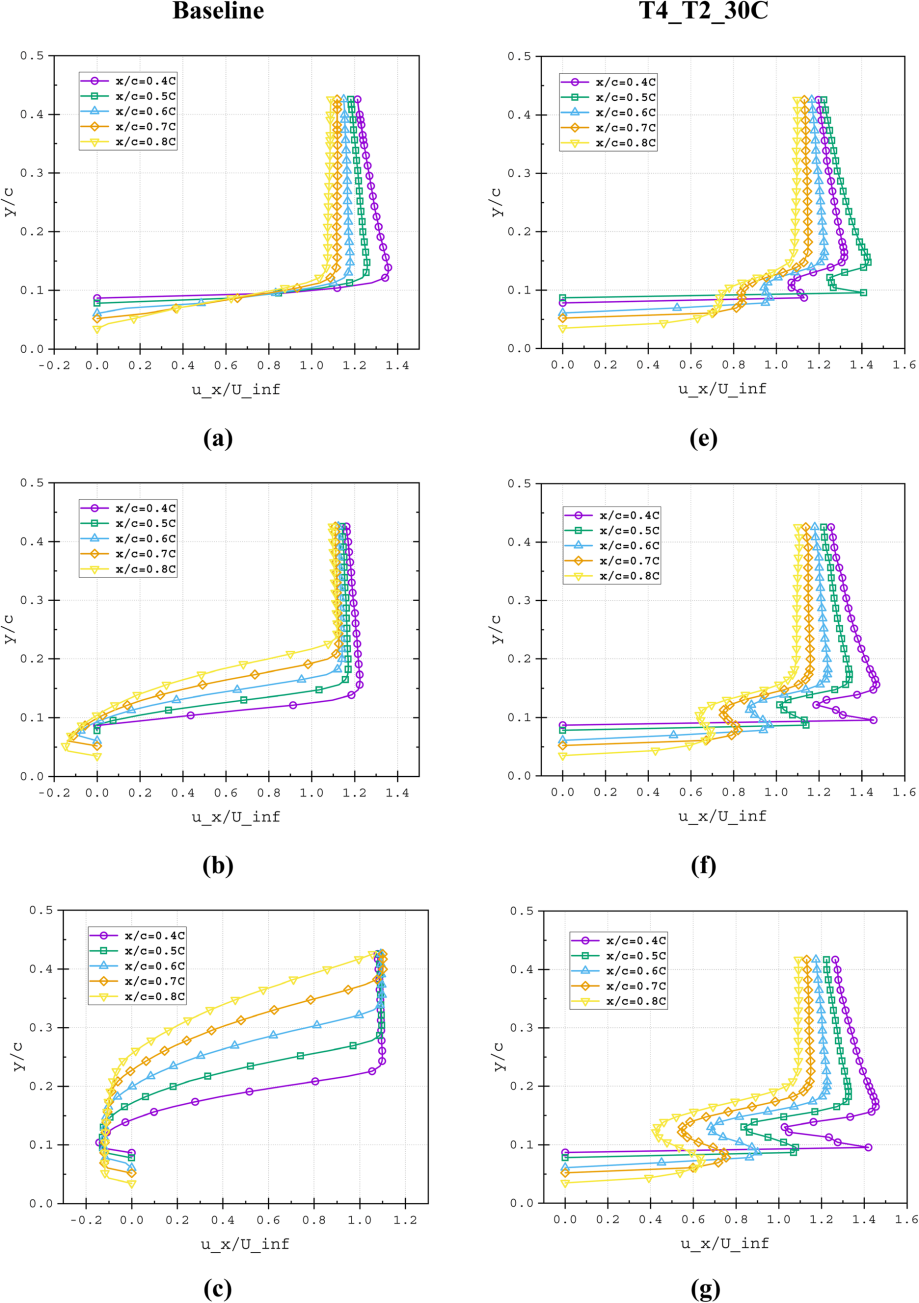

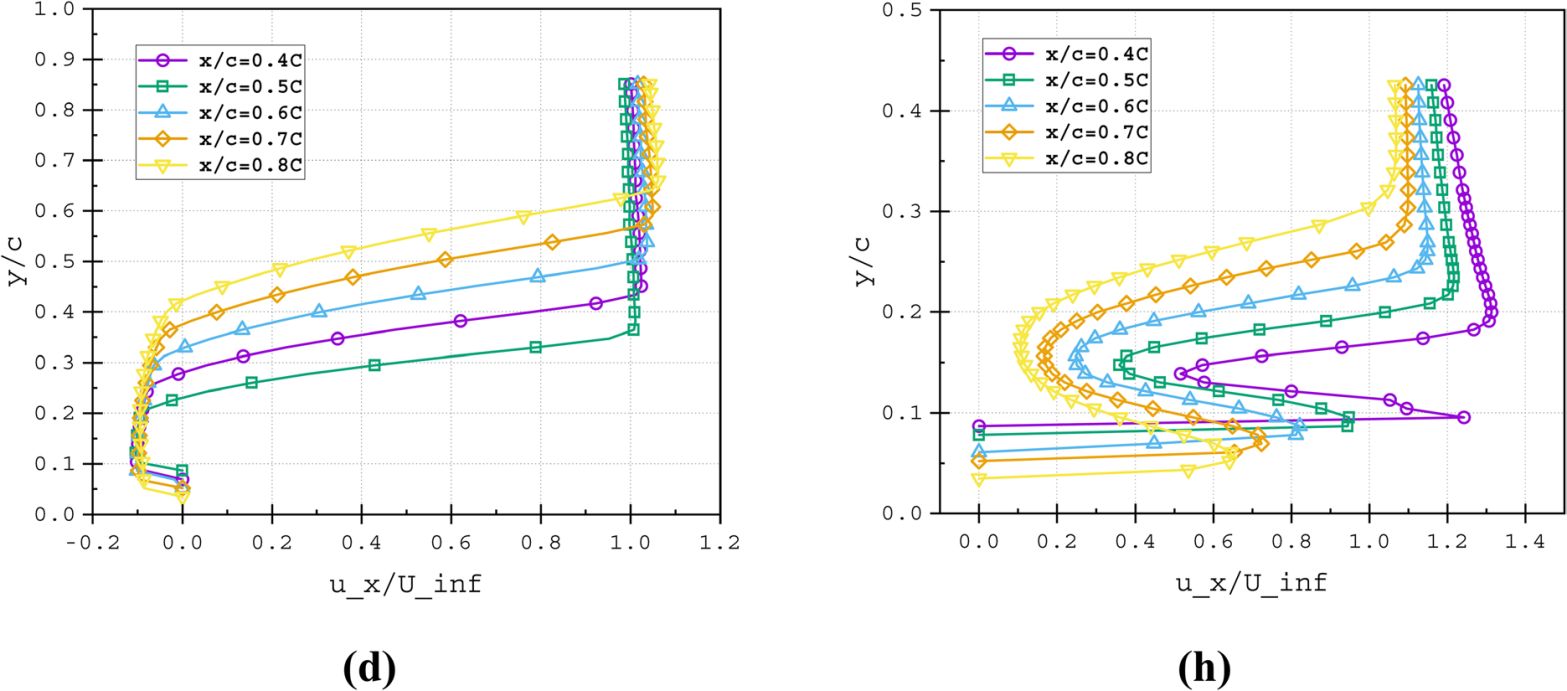
Non-dimensional velocity profile (*u*_*x*_*/U*_*∞*_) at various streamwise locations (*X/C*) of (**a**–**d**) Baseline and (**e**–**h**) T4_T2_30*C* slot configuration illustrated at (**a** and **e**) α = 15°, (**b** and **f**) α = 20°, (**c** and **g**) α = 25° and (**d** and **h**) α = 30°.

The characteristics of the flow field around the airfoil surface are crucial for identifying the behaviour of the airflow. Previously, in section "Mesh topology and grids", *C*_*F*_ was used to accurately identify the flow transition and flow detachment points. *C*_*F*_ is defined as the tangential resistance to the incoming flow. Additionally, the *C*_*F*_ indicates the state of the boundary layers. Zero *C*_*F*_ values suggest separation and reattachment of the flow; large turbulence in the flow can cause sudden drops in *C*_*F*_, meaning the detachment of the boundary layer, where the skin friction becomes insignificant and *C*_*F*_ approaches zero Fig. 8b. At AOAs of *α* = 5°, 10°, 15°, 20°, 25° and 30°, respectively flow separation for the baseline airfoil takes place at 98C, 90C, 65C, 25C, 12*C* and 8*C* of the chord length displayed in Fig. 8a and b. The T4_T2_30*C* effectively maintains the attached flow. The *C*_*F*_ values of the T4_T2_30*C* configuration barely drop along the suction surface, except at the stagnation point, at the tip of the wedge element and the bottom corner of the 3^rd^ element of the airfoil Fig. 19a–f. These regions correspond to the peaks in *C*_*P*_ Fig. 20a–f. These separation points are very crucial for constructing the slot, as the injected flow location has to be around or slightly before these points. Even if the slot location is too upstream of the separation point, and if the flow coming out of the slot has higher energy, then it can able to omit the separation (Fig. 21).

### Impact of split passage

**Fig. 22:**
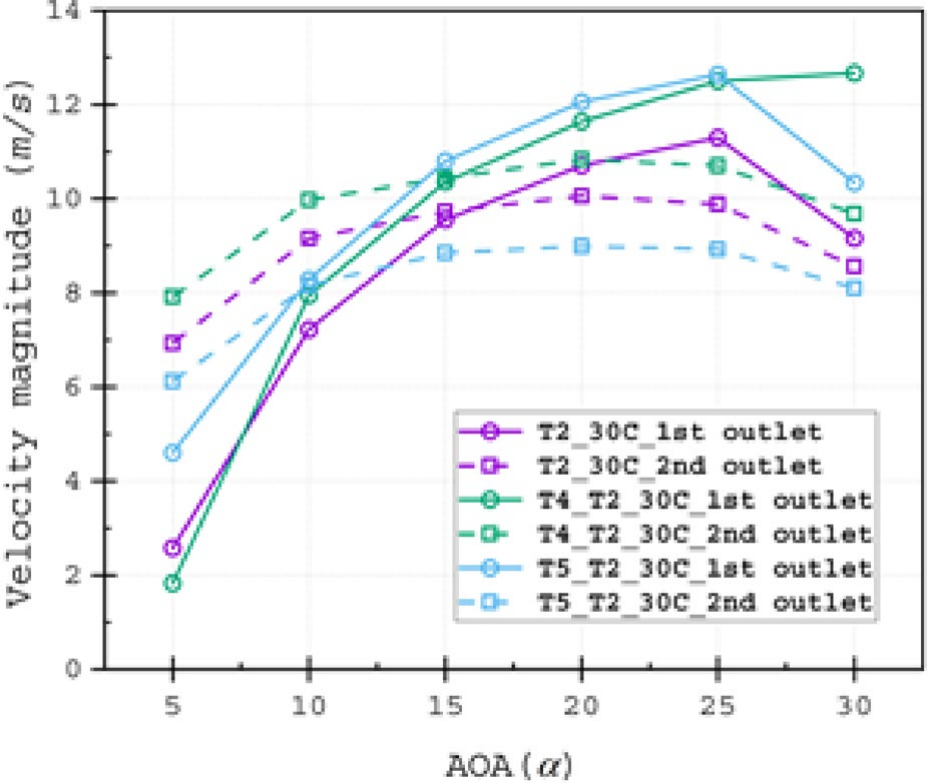
Average velocity magnitude at the outlet of both channels for T2_30C, T4_T2_30*C* and T5_T2_30*C* slot configurations.

The velocity through the split passage emerged as the dominant factor governing performance variations among slot configurations. To quantify this effect, we calculated the average fluid velocity at each slot outlet. Figure 22 reveals that while all three slot types exhibited similar velocity patterns at the 2^nd^ split across angles of attack (AOAs), their magnitudes followed a distinct hierarchy: Type 4 (widest outlet) > Type 2 > Type 5 (narrowest outlet). This trend directly correlates with outlet width and consequent mass flow rates, while confirming the 2^nd^ split’s negligible impact on flow anomalies.

Notably, at α = 5°, Type 5 demonstrated superior 1st split outlet velocity compared to Types 2 and 4. This phenomenon arises from: (1) vortex formation at the inlet corner, and (2) flow detachment at the second element’s tip edge, where wall contours of the third element redirect flow inertia toward the first split passage (Figs. 18 and 22). This behaviour is alleviated with increasing outlet width.

Above α = 25°, the stagnation point’s downstream shift reduces separation zones in Type 4 configurations. This promotes bubble formation near the third element’s inlet corner, channelling high-momentum flow into the first split passage (Fig. 18). Consequently, at α = 30°, Type 4 achieves greater first-split velocities than other configurations (Figs. 18 and 22). The enhanced momentum from this primary passage effectively counters adverse pressure gradients, delaying flow separation at high AOAs and significantly improving aerodynamic performance.

These findings establish the first split passage’s critical role in separation control, while demonstrating the double-slot system’s efficacy in maintaining attached flow and optimising efficiency across the operational AOA range.

This study demonstrates that optimal performance in double-split slotted airfoils necessitates sufficiently wide slot outlets to facilitate effective momentum transfer and flow control. The results reveal that constricted outflow passages function as flow obstructions, significantly compromising aerodynamic efficiency. Consequently, Type 5 slot configurations proved suboptimal for the present double-split design objectives, exhibiting systematically degraded performance across all operational parameters. These findings underscore the critical relationship between outlet geometry and flow management in slotted airfoil systems.

## Summary and conclusive remarks

The present study comprehensively analysed the effects of a novel double-split converged slot applied to a NACA 0018 airfoil on aerodynamic force coefficients using a detailed 2D numerical solution. A total of 28 slot configurations were evaluated at a Reynolds number of 250,000 across a wide angle of attack (AOA) range (*α* = 0°–35°).

- Results revealed that integrating double-split converged slots significantly enhanced aerodynamic performance and alleviated boundary layer separation. For the optimal T4_T2_30C configuration, a substantial improvement in the *C*_*L*_ by 118% and a reduction in the *C*_*D*_ by 49% were observed at *α* = 25° compared to the baseline case.
- At moderate to higher AOAs (10° ≤ *α* ≤ 30°), slot outlets positioned at 30% and 40% of the chord length (30*C* and 40*C*) performed optimally in suppressing flow separation.
- At high AOAs (*α* ≥ 15°), shorter wedge lengths and outlet locations nearer to the TE facilitated more fluid to pass through, while LE outlets were prone to failure due to the formation of thick vortices. Longer split channels with wider outlet openings further improved aerodynamic performance, particularly in the post-stall region, when positioned closer to the LE.
- The analysis highlighted that the first split of the slot had a greater impact on managing flow attachment and aerodynamic efficiency compared to the second split.
- The range factor (*R*_*f*_) proved effective in identifying optimal configurations, with the T4_T2_30C slot achieving the highest *R*_*f*_ value of 550.63—a 9% improvement over the baseline model.

These findings establish the double-split slotted airfoil as an effective passive flow control device, with potential applications in enhancing turbine performance. Future research should focus on optimising slot layouts with smaller AOA increments would improve the accuracy of the *R*_*f*_ assessment. Additionally incorporating these designs into VAWT systems, and conducting full-scale 3D numerical simulations and experimental studies should be appreciated work. Additionally, this kind of slot arrangement could effectively mitigate the adverse effects on turbine performance, such as dynamic stall, variable loads on the blades, noise generation, etc. The outcomes of this study are essential for advancing wind turbine blade design and contribute significantly to the development of sustainable and renewable energy technologies.

## Data Availability

The data that supports the findings of this study are available in the article.

## Abbreviations

2D: Two dimensional
3D: Three dimensional
AEP: Annual energy production
AFC: Active flow control
AOA: Angle of attack
CFD: Computation fluid dynamics
DES: Detached eddy simulation
DoE: Design of experiment
DU: Delft University
FDG: Flow deflecting gap
GIT: Grid independence test
HAWT: Horizontal axis wind turbine
LE: Leading edge
LoD: Lift-to-drag ratio
NACA: National advisory committee of aeronautics
NREL: National renewable energy laboratory
PFC: Passive flow control
RANS: Reynolds-averaged Navier–Stokes
RNG: Re-normalisation group
RSM: Response surface method
SB-VAWT: Straight blade-VAWT
TE: Trailing edge
TSR: Tip speed ratio
VAWT: Vertical axis wind turbine
VG: Vortex generator

## Symbols

*A*: The location of the centreline of the slot at the pressure surface from the leading edge
*B*: The location of the centreline of the slot at the suction surface from the leading edge
*C*: Chord length of Airfoil
*C*_*D*_: Drag coefficient
*C*_*F*_: Skin friction coefficient
*C*_*L*_: Lift coefficient
*C*_*L*_*/C*_*D*_: Lift-over-drag coefficient
*C*_*P*_: Power coefficient
*D*: Drag force
*K*: Turbulent kinetic energy
*L*: Lift force
*M*: Moment force
*P*: Static pressure at airfoil surface
*P*_*∞*_: Free stream static pressure
*R*_*e*_: Reynolds number
*R*_*f*_: Range factor
*U*_*∞*_: Free-stream velocity
*u*_*x*_*/U∞*: Non-dimensional velocity profile
*W*_*1*_: Slot inlet width
*W*_*2*_: Both slot outlet width
*X/C*: Non-dimensional chordwise length
*Y*: The vertical distance from the wedge tip (2nd element of the airfoil) to the location of the airfoil’s maximum bottom thickness
*Y* +: Nondimensional wall distance
*α*: Angle of attack
*Δα*: AOA range where the half of Maximum lift-over-drag coefficient situated
*ε*: Turbulent specific dissipation rate
*μ*: Dynamic viscosity
*ρ*: Density of fluid
*τ*_*w*_: Wall shear stress
*Ψ*: Slot inclination angle

## References

[1] Benmoussa, A. & Páscoa, J. C. Enhancement of a cycloidal self-pitch vertical axis wind turbine performance through DBD plasma actuators at low tip speed ratio. *Int J Thermofluids* **17**, 100258. 10.1016/j.ijft.2022.100258 (2023).

[2] Daraee, M. A. & Abbasi, S. A Novel approach to performance improvement of a VAWT using plasma actuators. *J. Clean. Prod.* **424**, 138876. 10.1016/j.jclepro.2023.138876 (2023).

[3] Guoqiang, Li. & Shihe, Yi. Large eddy simulation of dynamic stall flow control for wind turbine airfoil using plasma actuator. *Energy* **212**, 118753. 10.1016/j.energy.2020.118753 (2020).

[4] Guoqiang, L., Weiguo, Z., Yubiao, J. & Pengyu, Y. Experimental investigation of dynamic stall flow control for wind turbine airfoils using a plasma actuator. *Energy* **185**(1), 90–101 (2019).

[5] Hao, W., Bashir, M., Li, C. & Sun, C. Flow control for high-solidity vertical axis wind turbine based on adaptive flap. *Energy Convers. Manage.* **249**, 114845. 10.1016/j.enconman.2021.114845 (2021).

[6] Hao, W. & Li, C. Performance improvement of adaptive flap on flow separation control and its effect on VAWT. *Energy* **213**, 118809. 10.1016/j.energy.2020.118809 (2020).

[7] Liu, Q. et al. Effects of trailing-edge movable flap on aerodynamic performance and noise characteristics of VAWT. *Energy* **189**, 116271. 10.1016/j.energy.2019.116271 (2019).

[8] Elkhoury, M., Kiwata, T. & Aoun, E. Experimental and numerical investigation of a three-dimensional vertical-axis wind turbine with variable-pitch. *J. Wind Eng. Ind. Aerodyn.* **139**, 111–123. 10.1016/j.jweia.2015.01.004 (2015).

[9] Jain, P. & Abhishek, A. Performance prediction and fundamental understanding of small scale vertical axis wind turbine with variable amplitude blade pitching. *Renew. Energy* **97**, 97–113. 10.1016/j.renene.2016.05.056 (2016).

[10] Sagharichi, A., Zamani, M. & Ghasemi, A. Effect of solidity on the performance of variable-pitch vertical axis wind turbine. *Energy* **161**, 753–775. 10.1016/j.energy.2018.07.160 (2018).

[11] Rezaeiha, A., Montazeri, H. & Blocken, B. Active flow control for power enhancement of vertical axis wind turbines: leading-edge slot suction. *Energy* **189**, 116131. 10.1016/j.energy.2019.116131 (2019).

[12] Zhu, H., Hao, W., Li, C., Ding, Q. & Baihui, Wu. Application of flow control strategy of blowing, synthetic and plasma jet actuators in vertical axis wind turbines. *Aerosp. Sci. Technol.* **88**, 468–480. 10.1016/j.ast.2019.03.022 (2019).

[13] Akhter, M. Z., Ali, A. R., Jawahar, H. K., Omar, F. K. & Elnajjar, E. Enhanced energy extraction in small-scale wind turbines through slot-based passive blowing. *Energy Convers. Manag.*10.1016/j.ecmx.2023.100400 (2023).

[14] Baghdadi, M., Elkoush, S., Akle, B. & Elkhoury, M. Dynamic shape optimization of a vertical-axis wind turbine via blade morphing technique. *Renew. Energy* **154**, 239–251. 10.1016/j.renene.2020.03.015 (2020).

[15] Saravana Mohan et al., Design, Dynamics and Development of Upgraded Tiltable Wing associated Quadcopter through Advanced computational simulations incorporated Bottom-Up Approach. *Sci. Rep.* **15**(1), 12055. 10.1038/s41598-025-96036-0 (2025). 10.1038/s41598-025-96036-0 PMC11978954 40199905

[16] Liu, Q. et al. Aerodynamic and aeroacoustic performance assessment of a vertical axis wind turbine by synergistic effect of blowing and suction. *Energy Convers. Manage.* **271**, 116289. 10.1016/j.enconman.2022.116289 (2022).

[17] Moshfeghi, M., Ramezani, M. & Hur, N. Design and aerodynamic performance analysis of a finite span double-split S809 configuration for passive flow control in wind turbines and comparison with single-split geometries. *J. Wind Eng. Ind. Aerodyn.* **214**, 104654. 10.1016/j.jweia.2021.104654 (2021).

[18] Zhu, H., Hao, W., Li, C., Ding, Q. & Baihui, Wu. A critical study on passive flow control techniques for straight-bladed vertical axis wind turbine. *Energy* **165**, 12–25. 10.1016/j.energy.2018.09.072 (2018).

[19] Ahmad, M. & Zafar, M. H. Enhancing vertical axis wind turbine efficiency through leading edge tubercles: a multifaceted analysis. *Ocean Eng.* **288**, 116026. 10.1016/j.oceaneng.2023.116026 (2023).

[20] Anon.,. Researches on vortex generators applied to wind turbines: A review. *Ocean Eng.* **253**, 111266. 10.1016/j.oceaneng.2022.111266 (2022).

[21] De Tavernier, D., Ferreira, C., Viré, A., LeBlanc, B. & Bernardy, S. Controlling dynamic stall using vortex generators on a wind turbine airfoil. *Renew. Energy* **172**, 1194–1211. 10.1016/j.renene.2021.03.019 (2021).

[22] Gao, L., Zhang, H., Liu, Y. & Han, S. Effects of vortex generators on a blunt trailing-edge airfoil for wind turbines. *Renew. Energy* **76**, 303–311. 10.1016/j.renene.2014.11.043 (2015).

[23] Zhu, C. et al. Effects of the height and chordwise installation of the vane-type vortex generators on the unsteady aerodynamics of a wind turbine airfoil undergoing dynamic Stall. *Energy* **266**, 126418. 10.1016/j.energy.2022.126418 (2023).

[24] Zhu, C., Qiu, Y., Feng, Y., Wang, T. & Li, H. Combined effect of passive vortex generators and leading-edge roughness on dynamic stall of the wind turbine airfoil. *Energy Convers. Manage.* **251**, 115015. 10.1016/j.enconman.2021.115015 (2022).

[25] Aboelezz, A., Ghali, H., Elbayomi, G. & Madboli, M. A novel VAWT passive flow control numerical and experimental investigations: guided vane airfoil wind turbine. *Ocean Eng.* **257**, 111704. 10.1016/j.oceaneng.2022.111704 (2022).

[26] Arunvinthan, S., Nadaraja Pillai, S. & Cao, S. Aerodynamic characteristics of variously modified leading-edge protuberanced (LEP) wind turbine blades under various turbulent intensities. *J. Wind Eng. Ind. Aerodyn.* **202**, 104188. 10.1016/j.jweia.2020.104188 (2020).

[27] Gonçalves, A. N. C., Pereira, J. M. C. & Sousa, J. M. M. Passive control of dynamic stall in a H-darrieus vertical axis wind turbine using blade leading-edge protuberances. *Appl. Energy* **324**, 119700. 10.1016/j.apenergy.2022.119700 (2022).

[28] Johari, H., Henoch, C., Custodio, D. & Levshin, A. Effects of leading-edge protuberances on airfoil performance. *AIAA J.* **45**(11), 2634–2642. 10.2514/1.28497 (2007).

[29] Lositaño, I. C. & Danao, L. A. Steady wind performance of a 5 kW three-bladed H-rotor darrieus vertical axis wind turbine (VAWT) with cambered tubercle leading Edge (TLE) blades. *Energy* **175**, 278–291. 10.1016/j.energy.2019.03.033 (2019).

[30] Roy, S., Das, B. & Biswas, A. Effect of leading-edge protrusion shapes for passive flow control measure on wind turbine blades. *Ocean Eng.* **269**, 113688. 10.1016/j.oceaneng.2023.113688 (2023).

[31] Yan, Y., Avital, E., Williams, J. & Cui, J. Aerodynamic performance improvements of a vertical axis wind turbine by leading-edge protuberance. *J. Wind Eng. Ind. Aerodyn.* **211**, 104535. 10.1016/j.jweia.2021.104535 (2021).

[32] Charlesantonyraj Avaesprabha et al., Examinations of Power Extraction and Structural Integrity of a Nature Inspired Dual-Axis Wind Turbine under Diverse Operational Conditions. *Results in Engineering*, **27**, 105607. 10.1016/j.rineng.2025.105607 (2025).

[33] Wang, Z., Wang, Y. & Zhuang, M. Improvement of the aerodynamic performance of vertical axis wind turbines with leading-edge serrations and helical blades using CFD and Taguchi method. *Energy Convers. Manage.* **177**, 107–121. 10.1016/j.enconman.2018.09.028 (2018).

[34] Wang, Z. & Zhuang, M. Leading-edge serrations for performance improvement on a vertical-axis wind turbine at low tip-speed-ratios. *Appl. Energy* **208**, 1184–1197. 10.1016/j.apenergy.2017.09.034 (2017).

[35] Richard, P. R., Stephen, J. W. & Joseph, W. H. Particle image velocimetry investigation of the coherent structures in a leading-edge slat flow. *J Fluids Eng.*10.1115/1.4038091 (2017).

[36] Luo, D., Huang, D. & Sun, X. Passive flow control of a stalled airfoil using a microcylinder. *J. Wind Eng. Ind. Aerodyn.* **170**, 256–273. 10.1016/j.jweia.2017.08.020 (2017).

[37] Zhong, J., Li, J. & Liu, H. Dynamic mode decomposition analysis of flow separation control on wind turbine airfoil using leading−edge rod. *Energy* **268**, 126656. 10.1016/j.energy.2023.126656 (2023).

[38] Farhad, I. M. & Vijayaraghavan, K. The effects of aerofoil profile modification on a vertical axis wind turbine performance. *Energy* **80**, 20–31. 10.1016/j.energy.2014.11.034 (2015).

[39] Abdolahifar, A. & Karimian, S. M. H. A comprehensive three-dimensional study on darrieus vertical axis wind turbine with slotted blade to reduce flow separation. *Energy* **248**, 123632. 10.1016/j.energy.2022.123632 (2022).

[40] Akhter, M. Z., Jawahar, H. K., Omar, F. K. & Elnajjar, E. Performance characterization of a slotted wind turbine airfoil featuring passive blowing. *Energy Rep.* **11**, 720–735. 10.1016/j.egyr.2023.12.027 (2024).

[41] Belamadi, R., Djemili, A., Ilinca, A. & Mdouki, R. Aerodynamic performance analysis of slotted airfoils for application to wind turbine blades. *J. Wind Eng. Ind. Aerodyn.* **151**, 79–99. 10.1016/j.jweia.2016.01.011 (2016).

[42] Beyhaghi, S. & Amano, R. S. Multivariable analysis of aerodynamic forces on slotted airfoils for wind turbine blades. *J. Energy Res. Technol.* **141**(5), 051214. 10.1115/1.4042914 (2019).

[43] Bhavsar, H., Roy, S. & Niyas, H. Aerodynamic performance enhancement of the DU99W405 airfoil for horizontal axis wind turbines using slotted airfoil configuration. *Energy* **263**, 125666. 10.1016/j.energy.2022.125666 (2023).

[44] Hongpeng, L., Yu, W., Rujing, Y., Peng, X. & Qing, W. Influence of the modification of asymmetric trailing-edge thickness on the aerodynamic performance of a wind turbine airfoil. *Renew. Energy* **147**, 1623–1631. 10.1016/j.renene.2019.09.073 (2020).

[45] Mohamed, O. S., Ibrahim, A. A., Etman, A. K., Abdelfatah, A. A. & Elbaz, A. M. R. Numerical investigation of darrieus wind turbine with slotted airfoil blades. *Energy Convers. Manag.: X* **5**, 100026. 10.1016/j.ecmx.2019.100026 (2020).

[46] Narsipur, S., Brent P., and Michael S. CFD analysis of multielement airfoils for wind turbines. In: *30th AIAA Applied Aerodynamics Conference* (American Institute of Aeronautics and Astronautics, 2012).

[47] Ni, Z., Dhanak, M. & Tsung-chow, Su. Improved performance of a slotted blade using a novel slot design. *J. Wind Eng. Ind. Aerodyn.* **189**, 34–44. 10.1016/j.jweia.2019.03.018 (2019).

[48] Ramzi, M. Numerical study of long separation bubble on slotted thick airfoil. *PAMM*10.1002/pamm.201800411 (2018).

[49] Xie, Y. et al. Numerical and experimental investigation on the flow separation control of S809 airfoil with slot. *Math. Probl. Eng.* **2013**, e301748. 10.1155/2013/301748 (2013).

[50] Javaid, M. T. et al. Power enhancement of vertical axis wind turbine using optimum trapped vortex cavity. *Energy* **278**, 127808. 10.1016/j.energy.2023.127808 (2023).

[51] Roshan, Y., Milad, J. K., Nimvari, M. E. & Salarian, H. Performance improvement of darrieus wind turbine using different cavity layouts. *Energy Convers. Manage.* **246**, 114693. 10.1016/j.enconman.2021.114693 (2021).

[52] Ju, Y. P. & Zhang, C. H. Multi-point robust design optimization of wind turbine airfoil under geometric uncertainty. *Proc. Inst. Mech. Eng., Part A: J. Power Energy* **226**(2), 245–261. 10.1177/0957650911426540 (2012).

[53] Beyhaghi, S. & Amano, R. S. A parametric study on leading-edge slots used on wind turbine airfoils at various angles of attack. *J. Wind Eng. Ind. Aerodyn.* **175**, 43–52. 10.1016/j.jweia.2018.01.007 (2018).

[54] Ni, Z., Dhanak, M. & Tsung-chow, Su. Performance of a slotted hydrofoil operating close to a free surface over a range of angles of attack. *Ocean Eng.* **188**, 106296. 10.1016/j.oceaneng.2019.106296 (2019).

[55] Anon,. The handley page wing. *Aeronaut. J.* **25**(126), 263–289. 10.1017/S2398187300139817 (1921).

[56] Chandran et al., Design, aerodynamic performance and structural integrity investigations of aerofoil profiled Savonius vertical axis wind turbine. J *Braz. Soc. Mech. Sci. Eng.* **47**, 133. 10.1007/s40430-025-05413-3. (2025).

[57] Weick, F. E. The effect of multiple fixed slots and a trailing-edge flap on the lift and drag of a Clark y Airfoil. 12 (1933).

[58] Wenzinger, C. J., and Joseph A. S. The aerodynamic characteristics of a slotted clark y wing as affected by the auxiliary airfoil position (1932).

[59] Drela, M. Newton Solution of Coupled Viscous/Inviscid Multielement Airfoil Flows. In *21st Fluid Dynamics, Plasma Dynamics and Lasers Conference* (American Institute of Aeronautics and Astronautics, 1990).

[60] Drela, M. Design and Optimization Method for Multi-Element Airfoils.” In *Aerospace Design Conference *(American Institute of Aeronautics and Astronautics, 1993).

[61] Mdouki, R., and Abderrahmane G. Effects of slotted blading on secondary flow in highly loaded compressor cascade.” 8 (2013).

[62] Ibrahim, M., Alsultan, A., Shen, S. & Amano, R. S. Advances in horizontal axis wind turbine blade designs: Introduction of slots and tubercle. *J. Energy Res. Technol.* **137**(5), 051205. 10.1115/1.4030399 (2015).

[63] Moshfeghi, M. & Hur, N. Power generation enhancement in a horizontal axis wind turbine blade using split blades. *J. Wind Eng. Ind. Aerodyn.* **206**, 104352. 10.1016/j.jweia.2020.104352 (2020).

[64] Moshfeghi, M., Shams, S. & Hur, N. Aerodynamic performance enhancement analysis of horizontal axis wind turbines using a passive flow control method via split blade. *J. Wind Eng. Ind. Aerodyn.* **167**, 148–159. 10.1016/j.jweia.2017.04.001 (2017).

[65] Cui, G., Cao, Y., Yan, Y. & Wang, W. Hydrodynamic performance improvement on the hydrofoil using slotted configurations. *Ocean Eng.* **299**, 117350. 10.1016/j.oceaneng.2024.117350 (2024).

[66] Nia, B. B., Ja’fari, M., Ranjbar, A. R. & Jaworski, A. J. Passive control of boundary layer flow separation on a wind turbine airfoil using vortex generators and slot. *Ocean Eng* **283**, 115170. 10.1016/j.oceaneng.2023.115170 (2023).

[67] Battisti, L., Brighenti, A., Benini, E. & Raciti Castelli, M. Analysis of different blade architectures on small VAWT performance. *J. Phys: Conf. Ser.* **753**(6), 062009. 10.1088/1742-6596/753/6/062009 (2016).

[68] Feng, F. et al. Research on aerodynamic characteristics of straight-bladed vertical axis wind turbine with s series airfoils. *Int. J. Rotating Mach.* **2018**(1), 8350243. 10.1155/2018/8350243 (2018).

[69] Eggert, C. A., and Christopher L. R. CFD study of NACA 0018 airfoil with flow control. In *National Aeronautics and Space Administration (NASA)* (2017).

[70] Sengupta, A. R., Biswas, A. & Gupta, R. Studies of some high solidity symmetrical and unsymmetrical blade H-darrieus rotors with respect to starting characteristics, dynamic performances and flow physics in low wind streams. *Renew. Energy* **93**, 536–547. 10.1016/j.renene.2016.03.029 (2016).

[71] Ahmad, T., S. L. Plee, and J. P. Myers. “Fluent User’s Guide.” (2009).

[72] Shih, T. H., Liou, W. W. Shabbir, A. Yang, Z. and Zhu, J. A new K-epsilon eddy viscosity model for high reynolds number turbulent flows: Model development and validation. CMOTT-94-6 (1994).

[73] Rezaeiha, A., Montazeri, H. & Blocken, B. On the accuracy of turbulence models for CFD simulations of vertical axis wind turbines. *Energy* **180**, 838–857. 10.1016/j.energy.2019.05.053 (2019).

[74] Roache, P. J. Quantification of uncertainty in computational fluid dynamics. *Annu. Rev. Fluid Mech.* **29**, 123–160. 10.1146/annurev.fluid.29.1.123 (1997).

[75] Zishan, A. M., Ali, A. R., Jawahar, H. K., Omar, F. K. & Elnajjar, E. Performance enhancement of small-scale wind turbine featuring morphing blades. *Energy* **278**, 127772. 10.1016/j.energy.2023.127772 (2023).

[76] Jawahar, H. K., Qing A., and Mahdi A. Experimental and numerical investigation of aerodynamic performance of airfoils fitted with morphing trailing-edges. In *23rd AIAA/CEAS Aeroacoustics Conference*. American Institute of Aeronautics and Astronautics.

